# NsPEFs-enriched ADSCs-EVs alleviate osteoarthritis via RSPO3-mediated dual pro-chondrogenic and pro-M2 macrophage properties

**DOI:** 10.1016/j.bioactmat.2026.01.006

**Published:** 2026-01-17

**Authors:** Yushan Wang, Yingjie Gao, Zhiyan Cao, Mingjie Dong, Pengfei Shao, Hao Fan, Zijian Guo, Xiaoyong Hu, Wenxiang Cheng, Pengcui Li, Wei Zhang, Yi Feng, Panfeng Fu, Zigang Ge, Jiake Xu, Chuan Xiang

**Affiliations:** a Department of Orthopaedics, The Second Hospital of Shanxi Medical University, No. 382 of 51 Road, Taiyuan, Shanxi, China; b Shenzhen Institutes of Advanced Technology, Chinese Academy of Sciences, Shenzhen, China; c School of Medicine, Jiangsu Key Laboratory for Biomaterials and Devices, Southeast University, Nanjing, 210000, China; d Department of Respiratory and Critical Care, The Affiliated Hospital of Medical School of Ningbo University, Ningbo, China; e Department of Biomedical Engineering, Peking University, Beijing, 100871, China; f Shenzhen Univesity of Advanced Technology, Shenzhen, China; g School of Biomedical Sciences, The University of Western Australia, Perth, WA 6009, Australia

**Keywords:** Nanosecond pulsed electric fields (NsPEFs), Adipose-derived stem cells (ADSCs), Extracellular vesicles (EVs), Osteoarthritis, R-spondin 3 (RSPO3)

## Abstract

Osteoarthritis (OA) remains a debilitating joint disorder due to the lack of disease-modifying therapies that can simultaneously halt cartilage degradation and modulate the aberrant immune microenvironment. This study demonstrated the therapeutic potential of extracellular vesicles derived from adipose-derived stem cells preconditioned with nanosecond pulsed electric fields (NsPEFs-ADSCs-EVs). Administration of NsPEFs-ADSCs-EVs significantly attenuated OA progression, as indicated by alleviated cartilage degradation, and a marked shift in synovial macrophage from the pro-inflammatory M1 to the pro-reparative M2 phenotype.

Mechanistically, we discovered that NsPEFs-ADSCs-EVs, via surface-enriched ITGA4, activated the PI3K/Akt pathway to instruct the increased secretion of R-spondin 3 (RSPO3). We further unveiled a novel dual function of chondrocyte-derived RSPO3. It acted in an autocrine manner to enhance chondrocyte anabolism and in a paracrine manner to directly drive M2 macrophage polarization. The pro-M2 effect was specifically mediated through the activation of the LGR4/LRP6/β-catenin signaling axis in macrophages.

Collectively, this work elucidates a previously unrecognized paracrine axis wherein NsPEFs-engineered EVs deploy RSPO3 as a significant coordinator to synchronously promote cartilage regeneration and immune resolution. Our findings not only reveal RSPO3 as a promising therapeutic target but also establish the NsPEFs platform as a efficient strategy for generating functionally enhanced EVs, offering a novel cell-free strategy for OA therapy.

## Highlights

• NsPEFs-ADSCs-EVs as novel bio-active materials, exhibit high yield, homogeneity and enhanced bio-activity for OA treatment.
• NsPEFs-ADSCs-EVs instruct chondrocytes to transform into active signaling hubs by ITGA4-mediated inter-cellular communication.
• Chondrocyte-derived RSPO3 as a pivotal effector mediates the dual functionality, cartilage anabolism and M2 transition.
• The pro-M2 effect of RSPO3 is mechanistically operated through the LGR4/LRP6/β-catenin signaling axis in macrophages.
• A synergistic therapeutic strategy is proposed for OA by offering both the engineered EV platform and RSPO3 protein.

## Graphical abstract

**Figure:**
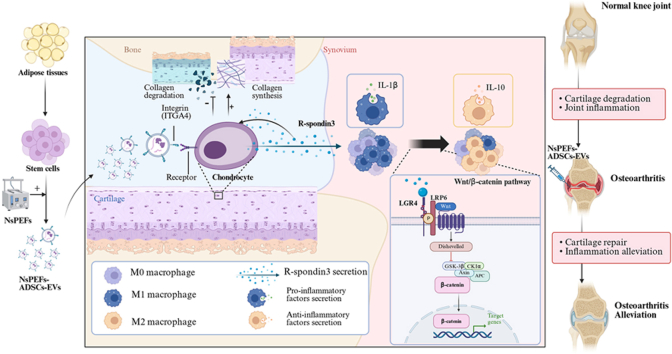

## 1 Introduction

Osteoarthritis (OA), the most prevalent degenerative joint disease, poses a formidable and unresolved clinical challenge due to the absence of therapies that can modify disease progression [1]. Contemporary understanding regards OA not as passive wear but as an active disorder of the entire joint, characterized by a maladaptive imbalance between persistent, low-grade inflammation and a failed intrinsic repair response [[2], [3], [4]]. Within this pathological microenvironment, a deleterious feedforward loop is established. Synovial pro-inflammatory macrophages drive chondrocyte dysfunction and extracellular matrix degradation, while damaged cartilage releases alarmins that perpetuate immune activation [[5], [6], [7], [8]]. Consequently, disrupting this crosstalk by simultaneously promoting anabolism and resolving inflammation has emerged as a pivotal therapeutic paradigm.

Adipose-derived stem cells-derived extracellular vesicles (ADSCs-EVs) have garnered significant interest as a promising cell-free nanotherapeutic platform, leveraging innate biocompatibility to deliver bioactive cargo [[9], [10], [11], [12]]. Preclinical studies support their potential in mitigating OA pathology [[13], [14], [15]]. However, two critical barriers impede clinical translation. First, scalable production of EVs with consistently enhanced and well-defined potency remains a challenge. While physical stimuli like nanosecond pulsed electric fields (NsPEFs) can augment EV release from mesenchymal stem cells as shown in studies focusing on yield and basic biological effects [[16], [17], [18]], it is unclear whether such preconditioning functionally enriches EVs with specific effector cargo, rather than merely increasing yield. Second, the precise mechanisms, particularly whether ADSCs-EVs regulate a coordinated and multi-cellular repair program via a defined molecular axis, are poorly defined. A key unanswered question is whether key EV-instructed factors can synchronously stimulate cartilage anabolism and anti-inflammatory macrophage polarization.

Here, we devised an optimized NsPPEFs stimulation protocol aimed not only at improving yield but also at steering cellular reprogramming [19]. Key parameters frequently neglected were finely controlled. The liquid volume and electrode gap were precisely aligned to ensure uniform electric field distribution, while a low-conductivity buffer was employed to reduce Joule heating and prevent nonspecific cellular damage. This refined approach enabled the administration of a low-energy pulsing regimen designed not to induce stress [16], but rather to redirect ADSCs toward a specific secretory phenotype. At the mechanism level, this study for the first time discovered that NsPEFs can specifically enrich integrin subunit alpha 4 (ITGA4)^2^ on the membrane surface of ADSCs-EVs. This is not only an increase in quantity but also a precise engineering of functional cargo, endowing EVs with the ability of active targeting and initiating specific downstream signals.

The search for key mediators capable of regulating the dual anabolic and immunomodulatory response led us to R-spondin 3 (RSPO3^3^), a secreted potentiator of the Wnt signaling pathway. RSPO3 remains mechanistically under-explored in OA. Its known signaling capacity through receptors such as Leucine-rich repeat-containing G-protein coupled receptor 4 (LGR4^4^) and its involvement in cell fate regulation position RSPO3 as a candidate to function as a central communication node [20,21]. Specifically, we hypothesize that RSPO3 may be a key EV-instructed factor that directly links cartilage repair signals to the reprogramming of synovial macrophages, thereby addressing the core therapeutic gap of synchronizing tissue anabolism with inflammation resolution.

Collectively, we propose that NsPEFs preconditioning can engineer ADSCs-EVs with not only enhanced yield but also functionally enriched cargo capable of reprogramming the OA joint via a central mediator. We demonstrate that NsPEFs-engineered ADSCs-EVs (NsPEFs-ADSCs-EVs), via their membrane protein ITGA4, activate PI3K/Akt signaling in chondrocytes to trigger RSPO3 secretion. The secreted RSPO3 then executes a dual function: acting locally to enhance cartilage matrix synthesis and operating as a paracrine signal to macrophages, where it activates the LGR4/LRP6^5^/β-catenin axis to drive M2 polarization. Our study thus moves beyond production enhancement to achieve functional EV engineering, unveils RSPO3 as a novel coordinator of tissue repair and immune regulation, and delineates a complete mechanistic axis from EV surface determinant to dual therapeutic outcomes.

## 2 Methods

### 2.1 Experimental animals

All the animal experimental protocols in this study were approved by the Experimental Animal Ethics Committee of the Second Hospital of Shanxi Medical University (DW2025011). Male Kunming mice (12-week-old) were purchased from Beijing Spirulina Biotechnology Co., Ltd. Mice were housed under standard specific-pathogen-free (SPF) conditions with a 12/12-h light/dark cycle and provided food and water ad libitum. The animal experiments followed the ARRIVE guidelines.

### 2.2 Cell culture

ADSCs: Mouse ADSCs were isolated from abdominal adipose tissue by enzymatic digestion with 0.25 mg/mL collagenase type I (Solarbio) for 50 min at 37 °C, following established protocols [22]. After filtration through a 100 μm strainer (NEST), cells were seeded at a density of 1 × 10^6^ cells/cm^2^ and maintained in α-MEM complete medium supplemented with 10 % fetal bovine serum (FBS), 1 % penicillin-streptomycin, and crucially, 10 ng/mL recombinant basic fibroblast growth factor (bFGF; PeproTech) [23]. The inclusion of bFGF is a well-documented strategy to sustain the proliferative capacity and stemness of MSCs during in vitro expansion [24,25]. Cells were cultured at 37 °C in a humidified atmosphere of 5 % CO_2_, with the medium replaced every 3 days. Upon reaching 80–90 % confluence, cells were passaged using a solution of TrypLE Express (ThermoFisher). More importantly, we strictly control the passage process, only passaging at a 1:3 ratio to avoid senescence and spontaneous differentiation caused by overgrowth. For all experiments in this study, only early-passage ADSCs (between passage 2 and 4) were used. The restriction to low passage numbers (≤P4) is a standard practice to minimize the risk of spontaneous differentiation and phenotypic drift, thereby ensuring the consistency and functionality of the cells utilized for extracellular vesicle production [24,26].

Bone marrow-derived macrophages (BMDMs): Bone marrow cells from the femur and tibia of mice were collected and resuspended in DMEM high-glucose medium containing 10 % FBS, 1 % antibiotics and 40 ng/mL macrophage colony-stimulating factor (PeproTech). The cells were seeded at a density of 2 × 10^6^ cells/mL and the medium was changed every 3.5 days.

Chondrocytes: Primary mouse chondrocytes were isolated from knee articular cartilage by sequential enzymatic digestion. Sacrifice young mice (typically under 1 week old) and aseptically dissect the limb joints. Remove surrounding tissues to isolate the translucent articular cartilage. Cut the cartilage into very small pieces (<1 mm^3^). Digest for 15–30 min at 37 °C using 0.25 % trypsin (Gibco). Wash thoroughly with PBS to remove the enzyme solution. Add type II collagenase (at a concentration of 0.1 %–0.2 %; thermofisher). Digest for 4–6 h at 37 °C in a constant-temperature shaker. The digested mixture was filtered through a 70 μm cell sieve to remove the undigested tissues. Centrifuge the filtrate and discard the supernatant. Resuspend the cells in complete medium (DMEM/F12), count them and evaluate the viability. The cells were cultured in a culture flask or plate with high-density inoculation. The ATDC5 murine chondrogenic cell line (passages 5–15, from Shanxi Medical University) was cultured in the same medium.

### 2.3 NsPEFs stimulation and EV isolation

ADSCs at a density of 1 × 10^6^ cells per 600 μL were resuspended in a low-conductivity buffer containing 10 % sucrose, 10 mM HEPES (ThermoFisher), and 1 mM KCl (pH 7.4) to ensure uniform electric field delivery and minimize joule heating. The cell suspension was transferred into a 0.4 cm-gap cuvette (Bio-Rad, 165–2088, USA). Cells were subjected to NsPEFs stimulation using 5 intermittent pulses (10 kV/cm field strength, 100 ns pulse width, 1 Hz frequency). The time interval between two pulses was 1 s with an overall processing time of 10 s [18]. This mild, multi-pulse paradigm was designed to modulate cellular signaling pathways with minimal acute cytotoxicity [27]. The total energy delivered to the cell suspension during this treatment was estimated to be approximately 14.4 mJ. Immediately after stimulation, cells were diluted in fresh serum-free α-MEM basic culture medium and cultured for 24 h to allow for EV secretion.

Conditioned medium was collected and subjected to differential centrifugation: 300×*g* for 10 min, 2000×*g* for 20 min, and 10,000×*g* for 30 min to remove cells, dead cells, and debris, respectively. The supernatant was added the Hieff Quick EV Isolation Kit (Yeasen Biotechnology) to further extract EVs and subjected to ultracentrifugation at 10,000×*g* for 70 min at 4 °C. The EV pellet was washed in PBS via a second ultracentrifugation and finally resuspended in 100–200 μL PBS. EV aliquots were stored at −80 °C and used within 2 weeks to ensure stability. Freeze-thaw cycles were avoided. Protein concentration was quantified by BCA assay, and the yield enhancement by NsPEFs was confirmed by parallel NTA and BCA measurements against control EVs from untreated ADSCs.

### 2.4 EV characterization

Nanoparticle Tracking Analysis (NTA): The EVs solution from two groups (n = 3 per group) were diluted with PBS to the appropriate concentration and analyzed using the Malvern Nanosight NS300 system. The particle concentration and size distribution were analyzed using NTA 3.4 software.

Transmission Electron Microscopy (TEM): EVs were deposited onto formvar-carbon coated grids, negatively stained with 2 % uranyl acetate, and imaged using a HT7800 transmission electron microscope.

Western blotting: EV markers (CD63, CD81, TSG101) and the negative marker Calnexin were detected in EV lysates (n = 3 per group).

Zeta potential:The Zeta potential of EVs was measured using the Malvern Zetasizer Nano ZS system at 25 °C to assess their surface charge and colloid stability. Each sample was measured three times.

### 2.5 Destabilization of the medial meniscus (DMM) model and in vivo interventions

Osteoarthritis was induced in the right knee of 12-week-old male Kunming mice via DMM surgery under isoflurane anesthesia. The mouse was placed in a supine position. The surgical area was disinfected with iodophor. On the inner side of the knee joint, a longitudinal skin incision about 5 mm long was made. The subcutaneous tissue and fascia were bluntly separated, and the joint capsule was longitudinally incised to expose and clearly see the medial meniscus and the lower part of the tibial plateau. Under the surgical microscope, identify the short ligament that connects the inner meniscus to the tibial plateau (the medial meniscus tibial ligament). Use a fine microscopical scissors to precisely cut this ligament. The joint capsule and skin incision were sutured layer by layer using absorbable sutures. The postoperative mice were housed individually until they regained consciousness. They were allowed to move freely and access food and water, and no external fixation was required.

At 7 weeks post-surgery, mice with established OA were randomly assigned to the following groups (n = 7–8/group) using a computer-generated block randomization sequence (block size = 4):OA + PBS, OA + Ctrl-ADSCs-EVs (50 μg/mL in 10 μL PBS, weekly), OA + NsPEFs-ADSCs-EVs (50 μg/mL in 10 μL PBS, weekly), OA + recombinant RSPO3 (R&D Systems; 40 ng/mL in 10 μL PBS, weekly) and Sham-operated control. For the RSPO3 knockdown experiment, a separate cohort of DMM mice received an intra-articular injection of AAV9-CRISPR/Cas9-sgRSPO3 or AAV9-CRISPR/Cas9-sgCtrl (1 × 10^9^ vg in 10 μL), followed by EVs treatment from week 7 post-surgery. All intra-articular injections were performed by a researcher blinded to the group allocation. Mice were euthanized at 11 weeks post-surgery for analysis.

### 2.6 Live imaging and safety assessment

To assess the in vivo distribution of EVs, some mice were injected with DiR-labeled EVs or an equal amount of free DiR into their joint cavities (n = 3 per group). Fluorescence signals were observed using a small animal live imaging system at 1, 6, 24 and 48 h after injection. At 72 h after the treatment, the main organs (heart, liver, spleen, lung, and kidney) were taken for ex vivo imaging to evaluate the systemic toxicity.

### 2.7 Histological and immunostaining analysis

Upon collection, joint specimens (n = 7 per group) were preserved in 4 % formaldehyde for a duration of 24 h, then rinsed with PBS. A decalcification process ensued, utilizing 10 % EDTA adjusted to pH 7.4, lasting 20 days. Paraffin-embedded tissue sections, 5 μm in thickness, underwent antigen retrieval, succeeded by the neutralization of endogenous peroxidase with 0.3 % hydrogen peroxide. These sections were then treated with goat serum (BOSTER) at 37 °C for an hour to block nonspecific binding, followed by an overnight incubation at 4 °C with specific antibodies. Post-incubation, the sections were washed with PBS and developed using a DAB staining kit (Zhongshan Golden Bridge). Quantitative analysis was conducted using ImageJ software. The Safranin O/Fast Green (SOFG) was carried out following the standard protocol. All histological sections were independently scored by two observers who were unaware of the group information, and the average score was calculated.

### 2.8 Immunofluorescence

Cells were cultured on cell slides (Biosharp) and immobilized with 4 % paraformaldehyde for half an hour. They were then permeabilized using Triton (Solarbio) for the same duration, after which they were blocked with goat serum (Solarbio) at 37 °C for 1 h. Primary antibodies were applied. The following day, the cells were exposed to fluorescent secondary antibodies for an hour, followed by nuclear staining with DAPI (BOSTER) for 30 min. In a similar immunohistochemical protocol mentioned before, tissue sections were sealed with an anti-fluorescence quenching solution (BOSTER) prior to examination.

### 2.9 Western blotting and co-immunoprecipitation (Co-IP)

Cells or tissues (n = 3 per group) were lysed in RIPA buffer. For western blotting, proteins were separated by SDS-PAGE, transferred to PVDF membranes, and probed with primary antibodies overnight at 4 °C. The antibodies used and the dilution ratios were as follows:Anti-INOS (1:800, Cohesion), Anti-Arginase 1 (1:800, BOSTER), Anti-LRP6 (1:800, BOSTER), Anti-Beta-catenin (1:800, BOSTER), Anti-CD163 (1:800, Abclonal), Anti-CD86 (1:800, BOSTER), Anti-LGR4 (1:800, Abclonal), Anti-IL-1β (1:800, BOSTER), Anti-IL-10 (1:1000, Bioss), Anti-MMP13 (1:800, BOSTER), Anti-COL2A1 (1:800, BOSTER), Anti-Histone H3 (1:1000, Nature Biosciences), Anti-Lamin A/C (1:1000, Nature Biosciences), Anti-Akt (1:1000, Nature Biosciences), Anti-pAkt (1:1000, Nature Biosciences), Anti-RSPO3 (1:1000, Abcam), Anti-CD63(1:800, BOSTER), Anti-CD81(1:800, BOSTER), Anti-TSG101(1:800, BOSTER), Anti-Calnexin(1:800, BOSTER). Blots were incubated with HRP-conjugated secondary antibodies (1:10000, Abcam) and visualized using a ChemiDoc Touch system (Bio-Rad).

For Co-IP, cell lysates were pre-cleared and incubated with anti-LGR4 antibody (Abclonal) or control IgG overnight, followed by incubation with Protein A/G beads. The immunoprecipitates were washed and analyzed by western blotting. Band intensities were quantified using ImageJ software.

### 2.10 RNA extraction and quantitative RT-PCR

Total RNA was extracted using the M5 Universal RNA Mini Kit (Mei5bio). cDNA was synthesized using the M5 Sprint qPCR RT Kit (Mei5bio). qPCR was performed on a QuantStudio 6 Flex system (Applied Biosystems) using M5 HiPer Real-time PCR Supermix (Mei5bio). Gene expression was normalized to Actb and calculated using the 2^∧^(-ΔΔCt) method. Primer sequences are listed in Table S1. For each sample, three technical replicates were set up.

### 2.11 Flow cytometry

Cells were blocked with anti-mouse CD16/32 and stained with fluorochrome-conjugated antibodies against CD86 (FITC, BioLegend) and CD206 (APC, BioLegend). Data were acquired on a CytoFLEX and analyzed using FlowJo software (Tree Star).

### 2.12 Proteomic analysis and single-cell RNA sequencing data analysis

Protein samples were separated by SDS-PAGE and visualized by Coomassie Blue R-250 staining. Protein digestion by trypsin was performed according to filter-aided sample preparation (FASP) procedure described by Matthias Mann. The digest peptides of each sample (n = 3 per group) were desalted on C18 Cartridges (Empore™ SPE Cartridges C18 (standard density), bed I.D. 7 mm, volume 3 ml, Sigma), concentrated by vacuum centrifugation and reconstituted in 40 μl of 0.1 % (v/v) formic acid. The peptide content was estimated by UV light spectral density at 280 nm using an extinctions coefficient of 1.1 of 0.1 % (g/l) solution that was calculated on the basis of the frequency of tryptophan and tyrosine in vertebrate proteins.

LC-MS/MS analysis was performed on a Q Exactive mass spectrometer (Thermo Scientific) that was coupled to Easy nLC (Proxeon Biosystems, now Thermo Fisher Scientific). The MS raw data for each sample were combined and searched using the MaxQuant 1.6.14 software for identification and quantitation analysis.

For enrichment analysis, the entire set of quantified proteins was used as the background dataset, with enrichment conducted via Fisher's exact test. To account for multiple comparisons, the Benjamini-Hochberg method was applied to adjust p-values, and functional categories or pathways were considered significant only if the adjusted p-value was below 0.05.

Single-cell data were obtained from the GEO database (GSE172500). Subsequently, the R package Seurat was used to normalize the single-cell data, and the Harmony algorithm was employed to correct for batch effects. The FindAllMarkers function was utilized in conjunction with classical macrophage marker annotations to identify cell types. The Scissor algorithm was applied to classify macrophages into different subgroups based on polarization-related characteristic markers. The Monocle algorithm was employed to construct a pseudotemporal trajectory of gene expression in macrophage subgroups.

### 2.13 Cellular uptake of EVs

The target cells were inoculated in confocal culture dishes. Once they adhered and grew to an appropriate density (such as 60–70 % fusion rate), the medium was replaced with fresh medium without phenol red. The experimental group was added with an equal amount of DiR-labeled NsPEFs-ADSCs-EVs or Ctrl-ADSCs-EVs (50 μg/mL). The cells were co-cultured at 37 °C and 5 % CO_2_ for 4–24 h. After the co-culture, the medium was discarded, and the cells were gently washed three times with pre-cooled PBS to remove the attached EVs on the surface. The cells were fixed with 4 % paraformaldehyde for 15 min and washed with PBS. Then, they were stained with DAPI for 10 min and washed with PBS. Images were taken using a laser confocal microscope. The fluorescence intensity differences within the cells of different groups (n = 3 per group) were statistically compared.

### 2.14 Statistical analysis

Data are presented as mean ± SEM. Statistical analyses were performed using GraphPad Prism 10. Normality and homogeneity of variance were assessed using Shapiro-Wilk and Brown-Forsythe tests, respectively. For comparisons between two groups, unpaired or paired two-tailed Student's t-tests were used. For multiple comparisons, one-way or two-way ANOVA followed by Tukey's or Sidak's post hoc test was applied. A p-value <0.05 was considered statistically significant.

## 3 Results

### 3.1 NsPEFs engineering generates ADSCs-EVs with enhanced yield, defined properties, and superior bioactivity in vitro

**Fig. 1:**
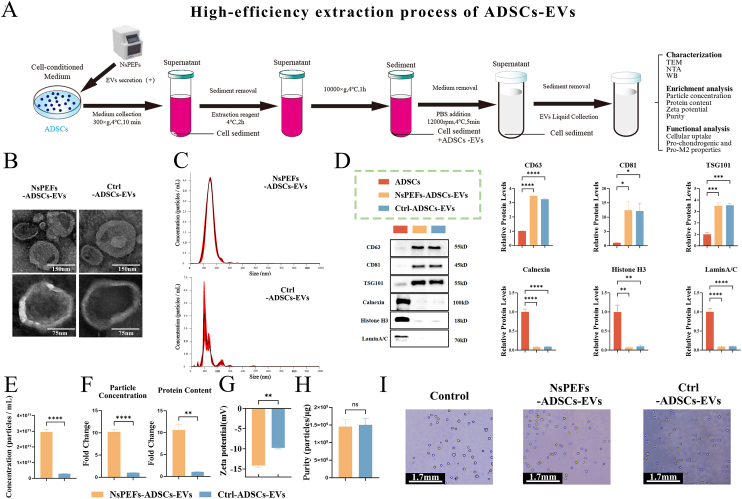
NsPEFs engineering boosts the production of ADSCs-EVs with superior yield and stability **A.**Schematic illustration of the high-efficiency extraction of extracellular vesicles (EVs) from adipose-derived stem cells (ADSCs) using nanosecond pulsed electric fields (NsPEFs). **B.**Representative transmission electron microscopy (TEM) images of isolated Ctrl-ADSCs-EVs and NsPEFs-ADSCs-EVs, showing characteristic cup-shaped morphology and bilayer membrane (scale bars: 150 nm and 75 nm). **C.**Nanoparticle tracking analysis (NTA) showing the particle size distribution of EVs (n = 3). **D.**Western blot (WB) analysis confirming the positive expression of EV-specific markers (CD81, CD63, TSG101) and the absence of the negative markers (Calnexin, Histone H3, LaminA/C). Quantification is shown on the right (n = 3). **E.**The particle concentration of EVs. **F.**NsPEFs stimulation significantly enhanced both yield and protein output compared to Ctrl-ADSCs-EVs. **G.**Zeta potential measurement indicating colloidal stability (n = 3). **H.**Purity assessment expressed as the particle-to-protein ratio ( × 10^9^ particles/μg). **I.**Viability of cells post-NsPEFs-ADSCs-EVs treatment assessed by trypan blue exclusion assay (scale bar: 1.7 mm). Data are presented as mean ± SEM from at least three independent experiments. Statistical significance was determined by unpaired two-tailed Student's t-test or one-way ANOVA with Tukey's post-hoc test. ∗P < 0.05, ∗∗P < 0.01, ∗∗∗P < 0.001, and ∗∗∗∗P < 0.0001; ns: not significant.

To establish an efficient source of therapeutic EVs, we employed NsPEFs [16,18] to precondition ADSCs (Fig. 1A and S1A). EVs isolated from both control (Ctrl-ADSCs-EVs) and NsPEFs-preconditioned ADSCs (NsPEFs-ADSCs-EVs) exhibited classic characteristics [28], as confirmed by cup-shaped morphology under transmission electron microscopy (TEM) (Fig. 1B), particle size distribution peaking around 100–160 nm via nanoparticle tracking analysis (NTA) (Fig. 1C), and positive expression of EV markers (CD81, CD63, TSG101) with absence of the negative markers (Calnexin, Histone H3, Lamin A/C) by western blotting (WB) [29] (Fig. 1D).

Critically, to substantiate the claim of “enrichment” by NsPEFs, we performed a side-by-side quantitative comparison. NsPEFs preconditioning significantly increased EV yield, with particle concentration elevated by approximately 10-fold and protein concentration elevated by approximately 10-fold compared to Ctrl-ADSCs-EVs (Fig. 1E and F). Notably, NsPEFs-ADSCs-EVs displayed a more uniform particle size distribution (Fig. 1C) and a significantly higher absolute zeta potential compared to Ctrl-ADSCs-EVs (Fig. 1G), indicating a stronger electrostatic repulsion and potentially superior colloidal stability that may contribute to the preservation of their bioactivity during storage and application. Importantly, TEM images confirmed that both preparations consisted of intact vesicles with a bilayer membrane structure, and no signs of excessive cellular debris or apoptotic bodies were observed (Fig. 1B). The purity of the EV preparations, as determined by the ratio of particle concentration to protein concentration, was 1.4 × 10^9^ particles/μg for NsPEFs-ADSCs-EVs and 1.5 × 10^9^ particles/μg for Ctrl-ADSCs-EVs, indicating a high degree of enrichment for vesicular structures and minimal co-isolation of non-vesicular contaminants (Fig. 1H). The trypan blue staining showed that both types of EVs had good biocompatibility (Fig. 1I).

**Fig. 2:**
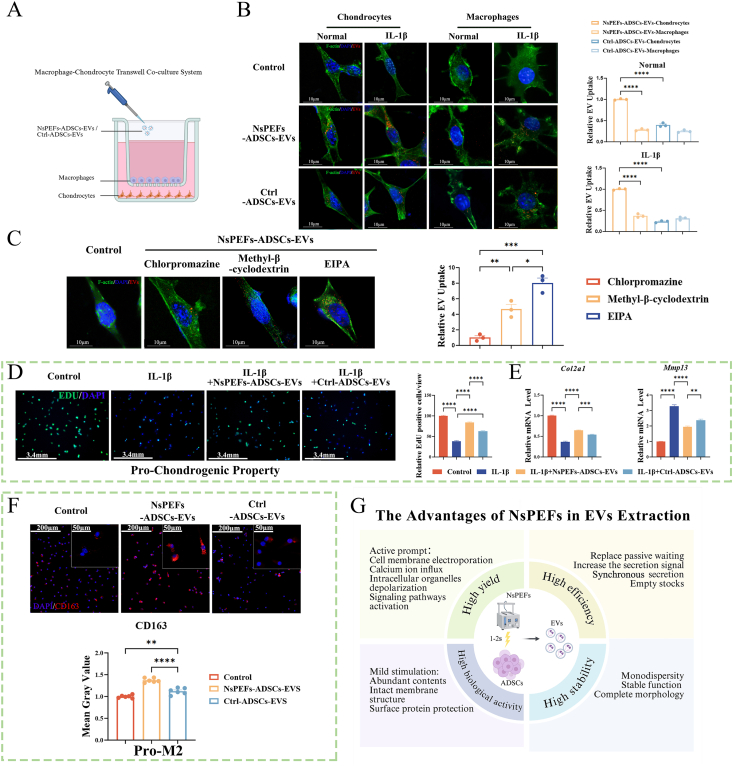
NsPEFs-ADSCs-EVs potentiate chondroprotective and immunomodulatory activities in vitro **A.**Schematic of the macrophage-chondrocyte transwell co-culture system. **B.**Cellular uptake of DiR-labeled EVs (red) by chondrocytes and macrophages in co-culture system, with nuclei stained by DAPI (blue). Quantification of fluorescence intensity is shown (scale bar: 10 μm; n = 3). **C.**Cellular uptake of DiR-labeled EVs (red) by chondrocytes pretreated with endocytosis inhibitors, chlorpromazine (clathrin-mediated), methyl-β-cyclodextrin (caveolae-mediated), and EIPA (macropinocytosis). Quantification is shown (scale bar: 10 μm; n = 3). **D, E.**NsPEFs-ADSCs-EVs exhibit enhanced pro-chondrogenic activity. **(D)** EdU proliferation assay (with quantitative analysis, right panel) and **(E)** qPCR analysis of anabolic (*Col2a1*) and catabolic (*Mmp13*) genes showed that NsPEFs-ADSCs-EVs (50 μg/mL) promoted chondrocyte proliferation and matrix homeostasis more effectively than Ctrl-ADSCs-EVs (n = 3; scale bar: 3.4 mm). **F.**NsPEFs-ADSCs-EVs drive M2 macrophage polarization more effectively. Immunofluorescence staining for the M2 macrophage marker CD163 (red) following EVs treatment, with nuclei counterstained by DAPI (blue). Quantification of CD163^+^ cells is shown (scale bars: 50 μm for inset, 200 μm for overview; n = 6). **G.**Schematic summary of the "4H" advantages of the NsPEFs approach: High yield, High efficiency, High biological activity, and High stability. Data are presented as mean ± SEM from at least three independent experiments. Statistical significance was determined by one-way ANOVA with Tukey's post-hoc test. ∗P < 0.05, ∗∗P < 0.01, ∗∗∗P < 0.001, and ∗∗∗∗P < 0.0001; ns: not significant.

We next assessed whether these advantages in terms of representation translated to improved functional engagement with target cells. Using DIR-labeled EVs and macrophage-chondrocyte co-culture system (Fig. 2A), we demonstrated that both EV types were effectively internalized by chondrocytes. However, NsPEFs-ADSCs-EVs exhibited a significantly higher uptake efficiency and a stronger chondrocyte targeting property, as quantified by increased intracellular fluorescence intensity in chondrocytes with the same concentration (50 μg/mL) and the same intervention duration (12 h; Fig. 2B and S1B). This enhanced cellular entry correlated with superior bioactivity. Pharmacological inhibition experiments revealed that the EVs uptake by chondrocytes was significantly reduced by chlorpromazine, an inhibitor of clathrin-mediated endocytosis, and partly reduced by Methyl-β-cyclodextrin. But the uptake was not significantly reduced by the inhibitor of macropinocytosis [[30], [31], [32]] (Fig. 2C). This indicates that clathrin-mediated endocytosis is the primary route for NsPEFs-ADSCs-EVs internalization into chondrocytes, which may result from the binding of surface ligands on NsPEFs-ADSCs-EVs to the membrane receptors of chondrocytes, and has certain specificity. At an optimized dose of 50 μg/mL, NsPEFs-ADSCs-EVs promoted chondrocyte proliferation more effectively than Ctrl-ADSCs-EVs, as shown by EdU and CCK-8 assays (Fig. 2D and S1C). In IL-1β-stimulated chondrocytes, NsPEFs-ADSCs-EVs more potently reverse the catabolic state by upregulating the anabolic gene (*Col2a1*) and downregulating the catabolic gene (*Mmp13*) (Fig. 2E).

Finally, to evaluate the immunomodulatory potential, immunofluorescence staining was performed using the M2 marker. The results showed that NsPEFs-ADSCs-EVs induced a significantly stronger M2 polarization response than Ctrl-ADSCs-EVs in the co-culture system, evidenced by higher M2 gene expression (CD163) (Fig. 2F). Collectively, these data establish that NsPEFs engineering not only boosts EV production but also functionally modifies EVs with enhanced stability, target cell internalization, and dual chondroprotective and immunomodulatory activities in vitro. We summarized the key advantages of the NsPEFs approach as the "4 H″, High yield, High efficiency, High biological activity, and High stability, in a schematic diagram (Fig. 2G).

### 3.2 NsPEFs-enriched ADSCs-EVs ameliorate disease progression in a post-traumatic OA model

**Fig. 3:**
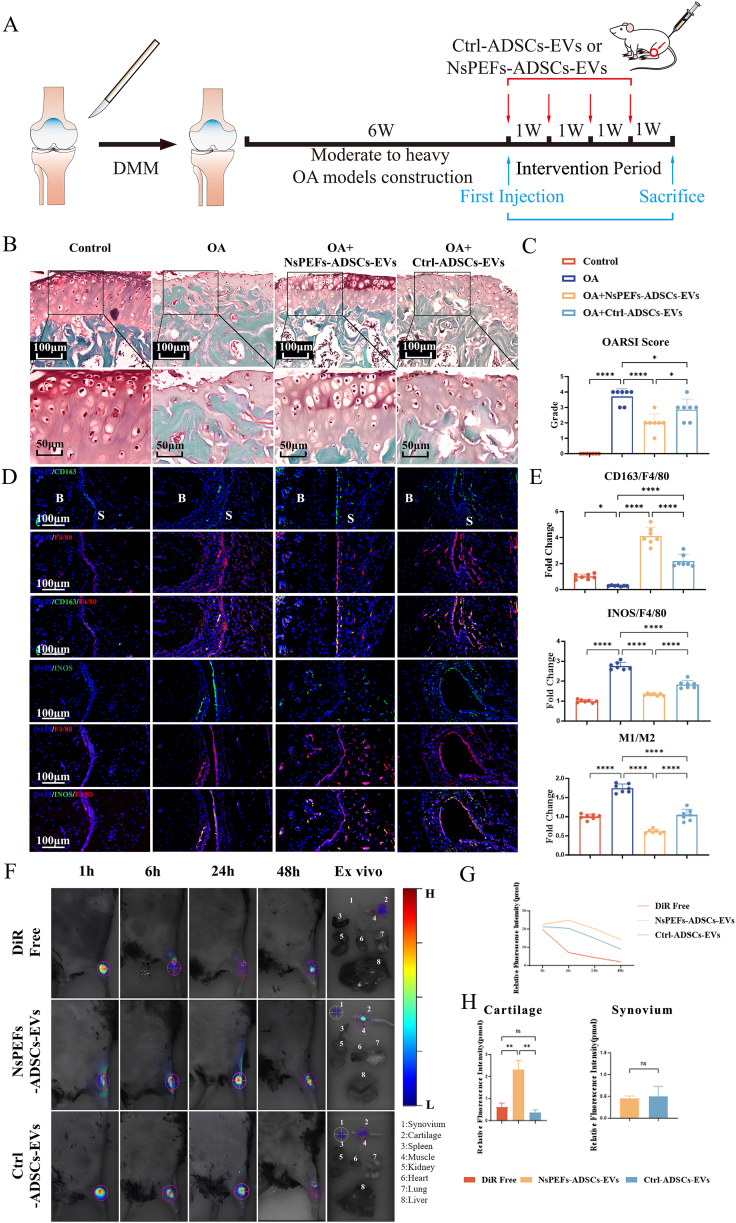
NsPEFs-enriched ADSCs-EVs ameliorate disease progression in a post-traumatic OA model **A.** Experimental timeline of the destabilization of the medial meniscus (DMM) model and subsequent therapeutic intervention with weekly intra-articular injections of NsPEFs-ADSCs-EVs (50 μg/mL in 10 μL PBS) or Ctrl-ADSCs-EVs (50 μg/mL in 10 μL PBS), starting at 6 weeks post-surgery when moderate-to-severe OA was established. **B, C.** NsPEFs-ADSCs-EVs exihibit a superior ability of cartilage protection. **(B)** Representative Safranin O-Fast Green (SOFG) staining of knee joint sections (scale bars: 100 μm and 50 μm) and **(C)** corresponding quantitative OARSI scores show that NsPEFs-ADSCs-EVs more effectively mitigate cartilage degradation and proteoglycan loss compared to the Ctrl-ADSCs-EVs and OA group (n = 7). **D, E.** NsPEFs-ADSCs-EVs potently remodel the synovial immune microenvironment. **(D)** Representative immunofluorescence images of synovial tissue co-stained for the pan-macrophage marker F4/80 (red) with the M2 marker CD163 (green) or the M1 marker iNOS (green). Nuclei are stained with DAPI (blue) (Scale bar: 100 μm). ‘S’ indicates synovial tissue area; ‘B’ indicates bone tissue area. **(E)** Quantification of the fluorescence intensity ratio demonstrated that NsPEFs-ADSCs-EVs significantly decreased the M1/M2 macrophage ratio, promoting a pro-repair synovial environment (n = 7). F-H. In vivo biodistribution and retention of EVs. Representative fluorescence molecular tomography (FMT) images of mouse knee joints at indicated time points after a single intra-articular injection of DiR-labeled NsPEFs-ADSCs-EVs or Ctrl-ADSCs-EVs. Color scale indicates fluorescence intensity. Ex vivo fluorescence images of major organs and major tissues in knee joints harvested 72 h post-injection, confirmed prolonged local retention in NsPEFs-ADSCs-EVs group and uncaptured systemic distribution to distant organs (n = 3). Data are presented as mean ± SEM. Statistical significance was determined by one-way ANOVA with Tukey's post-hoc test for multiple comparisons. ∗P < 0.05, ∗∗P < 0.01, ∗∗∗P < 0.001, ∗∗∗∗P < 0.0001.

Having established the superior in vitro properties of NsPEFs-ADSCs-EVs, we evaluated their therapeutic efficacy in a well-established post-traumatic OA model induced by destabilization of the medial meniscus (DMM) in young male mice [33] (Fig. 3A). This model was chosen for its high reproducibility in recapitulating the sequential events of cartilage degradation and synovial inflammation relevant to human PTOA [24,[34], [35], [36], [37], [38], [39]]. Mice received intra-articular injections of PBS (10 μL), Ctrl-ADSCs-EVs (50 μg/mL in 10 μL), or NsPEFs-ADSCs-EVs (50 μg/mL in 10 μL) weekly for 4 weeks post-surgery. The in vivo dose was extrapolated from in-vitro efficacy data, previous EV therapy studies in murine OA models [[39], [40], [41], [42], [43], [44], [45]] and initial in-vivo dose exploration experiments (Fig. S1C and S2A, B).

Histopathological evaluation using Safranin O-Fast Green (SOFG) staining demonstrated that both EV treatments attenuated cartilage degradation, but the NsPEFs-ADSCs-EVs group exhibited the most pronounced protective effect, showing significantly lower OARSI scores, greater cartilage thickness, and higher proteoglycan content (Fig. 3B and C). Strikingly, immunofluorescence analysis of synovial tissues revealed that NsPEFs-ADSCs-EVs treatment more effectively rebalanced the macrophage population, significantly increasing the ratio of CD163^+^ M2 macrophages while reducing iNOS^+^ M1 macrophages (Fig. 3D and E).

To understand the in-vivo metabolism and distribution basis for this enhanced efficacy, we performed live imaging after intra-articular injection of DiR-labeled EVs. NsPEFs-ADSCs-EVs demonstrated significantly prolonged retention within the knee joint compared to Ctrl-ADSCs-EVs, as quantified by fluorescence intensity over time (Fig. 3F and G). Crucially, the signal from free DiR dye cleared rapidly, confirming that the observed retention was a property of the ADSCs-EVs. Ex vivo imaging of harvested joints and major organs at 72 h confirmed the superior local accumulation of NsPEFs-ADSCs-EVs, especially in cartilage, and revealed undetectable systemic distribution to organs like the liver and spleen, indicating a favorable safety profile (Fig. 3F–H). Furthermore, immunofluorescence analysis of joint sections demonstrated the co-localization of EVs with both cartilage layer and synovial tissues, providing direct anatomical support for their engagement with the target cells. Among them, NsPEFs-ADSCs-EVs demonstrated greater cartilage targeting ability (Fig. S2C).

Collectively, these results demonstrate that NsPEFs-ADSCs-EVs not only possess superior in-vitro bioactivity but also exhibit enhanced joint retention and targeted delivery in vivo, culminating in significantly improved therapeutic outcomes for cartilage layer protection and pro-repair synovial immune environment.

### 3.3 NsPEFs-enriched ADSCs-EVs stimulate chondrocytes to secrete RSPO3, a key mediator of pro-chondrogenic effects

**Fig. 4:**
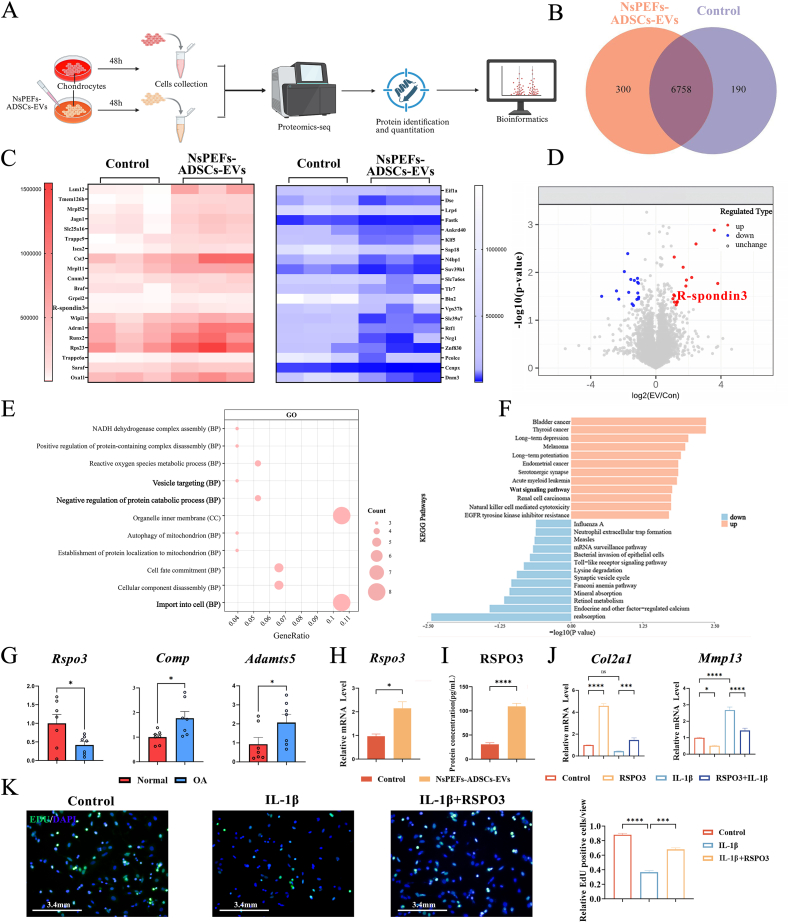
NsPEFs-enriched ADSCs-EVs stimulate chondrocytes to secrete RSPO3, a key mediator for the pro-chondrogenic effect **A.**Experimental schematic for the proteomic analysis of chondrocytes following NsPEFs-ADSCs-EVs treatment. **B-D.**Proteomic profiling identifies RSPO3 as a highly up-regulated protein. **(B)** Venn diagram shows the numbers of differentially expressed proteins (DEPs) in chondrocytes after EVs intervention (n = 3). **(C)** The heat map of significantly up-regulated (red) and down-regulated (blue) DEPs, with RSPO3 highlighted. **(D)** The volcano plot visualizes the DEPs, further confirming RSPO3 as one of the most significantly upregulated proteins. **E, F.** Functional enrichment analysis of DEPs. **(E)** Gene Ontology (GO) terms and **(F)** Kyoto Encyclopedia of Genes and Genomes (KEGG) pathway analyses show the significant enrichment for processes related to vesicle targeting, negative regulation of protein catabolic process and Wnt signaling pathway. **G.**Clinical relevance of RSPO3 in human OA. Re-analysis of a human transcriptomic dataset (E-MTAB-4304) confirms that *Rspo3* expression is significantly down-regulated in damaged cartilage of OA patients compared to intact human cartilage. *Comp* and *Adamts5* are shown as negative makers for cartilage anabolism (n = 7). **H.**Experimental validation of RSPO3 expression and secretion. qPCR analysis confirms that NsPEFs-ADSCs-EVs significantly up-regulate the level of *Rspo3* mRNA in chondrocytes (n = 6). **I.**ELISA confirms a consequent increased level of RSPO3 secretion (n = 6). **J, K.**Functional validation of RSPO3. **(**K**)** EdU proliferation assay (with quantitative analysis, right panel, scale bar: 3.4 mm) and **(**J**)** qPCR analysis demonstrate that recombinant RSPO3 protein directly promote the proliferation and anabolism in chondrocyte by up-regulating *Col2a1* and down-regulating *Mmp13* (n = 6). Data are presented as mean ± SEM from at least three independent experiments. Statistical significance was determined by unpaired two-tailed Student's t-test or one-way ANOVA with Tukey’ s post-hoc test. ∗P < 0.05, ∗∗P < 0.01, ∗∗∗P < 0.001, and ∗∗∗∗P < 0.0001.

To identify the pivotal molecular mediators of NsPEFs-ADSCs-EVs responsible for the dual pro-chondrogenic and pro-M2 properties, we performed quantitative proteomics on chondrocytes following NsPEFs-ADSCs-EVs treatment (Fig. 4A). This analysis revealed 490 differential proteins (Fig. 4B). Notably, R-spondin 3 (RSPO3), a secreted agonist of the Wnt/β-catenin pathway, was among the most prominently upregulated proteins (Fig. 4C and D). Gene Ontology (GO) analysis indicated enrichment in processes related to vesicle targeting and protein metabolism, while Kyoto Encyclopedia of Genes and Genomes (KEGG) pathway analysis specifically highlighted a significant enrichment for the Wnt pathway, positioning RSPO3 as a strategically relevant candidate (Fig. 4E and F). The clinical relevance of RSPO3 in OA was substantiated by re-analyzing a human transcriptomic dataset (E-MTAB-4304) [46], which confirmed that RSPO3 expression was significantly downregulated in damaged OA cartilage versus intact human cartilage while catabolism indicators (Comp and Adamts5) significantly increased.(Fig. 4G). We then validated in chondrocytes that NsPEFs-ADSCs-EVs treatment significantly increased both intracellular and secreted levels of RSPO3 using qPCR, WB, and ELISA (Fig. 4H, I and Fig. S3A).

Having identified RSPO3 as an EVs-induced, OA-relevant factor, we next determined whether it was functionally responsible for the observed chondroprotection. Indeed, recombinant RSPO3 protein [21] (40 ng/mL, a concentration optimized based on dose-response curves; Fig. S3B) potently promoted chondrocyte proliferation and anabolism in vitro, upregulating *Col2a1* and downregulating *Mmp13* expression in chondrocytes (Fig. 4J and K). Conversely, neutralization of secreted RSPO3 with Rosmantuzumab [47], a specific antibody, significantly inhibited the pro-chondrogenic effect of NsPEFs-ADSCs-EVs (Fig. S3C). These gain- and loss-of-function experiments establish that RSPO3 as a central effector molecule secreted by NsPEFs-ADSCs-EVs-treated chondrocytes, is necessary for mediating the pro-chondrogenic activities.

### 3.4 RSPO3 directly promotes M2 macrophage polarization through the LGR4/LRP6/β-catenin signaling axis

#### 3.4.1 RSPO3 acts as a direct paracrine signal to instruct M2 polarization

**Fig. 5:**
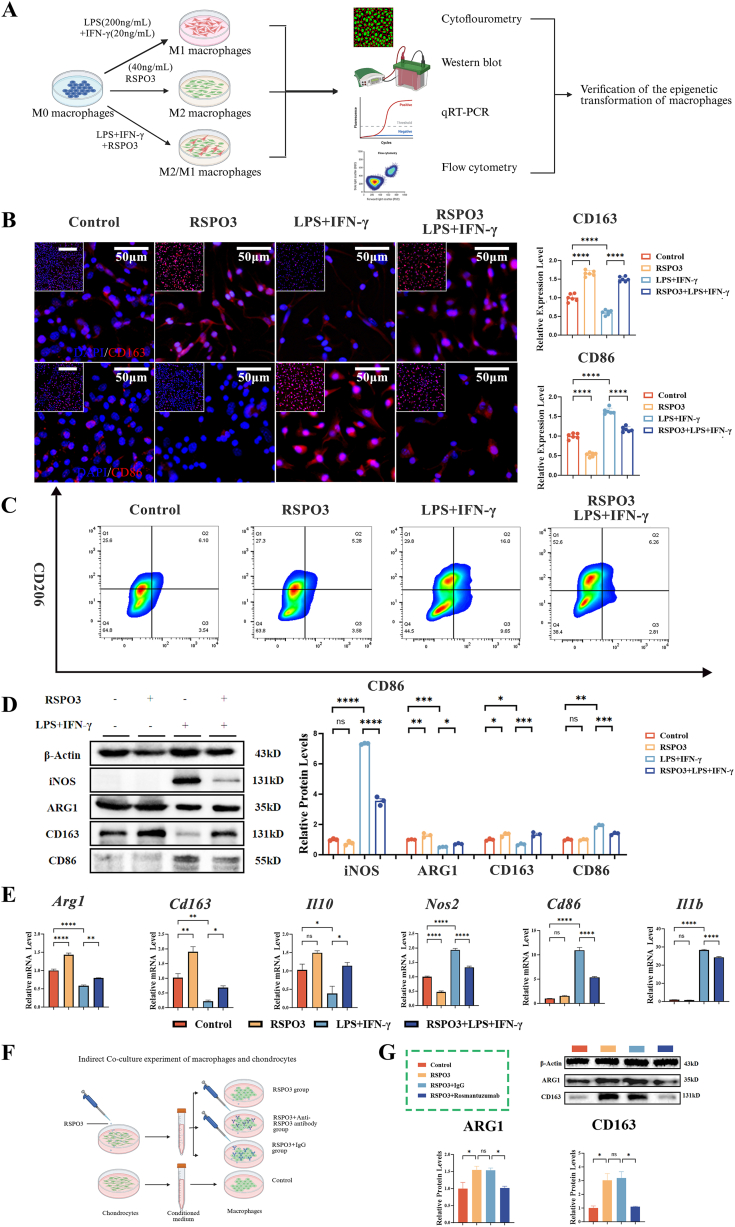
RSPO3 acts as a direct paracrine signal to instruct M2 polarization. **A.**Experimental schematic of RSPO3 treatment on bone marrow-derived macrophages (BMDMs) in the context of LPS/IFN-γ-induced M1 polarization. **B.**Representative immunofluorescence images of macrophages stained for the M2 marker CD163 (red) and the M1 marker CD86 (red), show that RSPO3 treatment promote the M2 transition while inhibiting the M1 transition induced by LPS/IFN-γ. Nuclei are stained with DAPI (blue). Scale bars: 50 μm (overview) and 200 μm (inset). (n = 6) **C.**Flow cytometric analysis of macrophages using antibodies against CD206 (M2), CD86 (M1), and F4/80 (pan-macrophage) confirms that RSPO3 significantly increases the ratio of CD206^+^ M2 macrophages and decreases the ratio of CD86^+^ M1 macrophages. **D**.WB analysis of key M1 (iNOS, CD86) and M2 (ARG1, CD163) markers, demonstrates that protein-level regulation of RSPO3 in macrophages aligns with the observed phenotypic shift. β-actin serves as a loading control (n = 3). **E**.qPCR analysis of M1 (*NOS2, Cd86, Il1b*) and M2 (Arg*1, Cd163, Il10*) genes in macrophages confirms that RSPO3 treatment reprograms macrophages towards a pro-repair, anti-inflammatory M2 state (n = 6). **F**.Schematic of the indirect co-culture system of macrophages and chondrocytes. **G.**WB analysis of key M2 markers demonstrates that the pro-M2 effect induced by RSPO3 is abolished when RSPO3 is neutralized (n = 3). Data are presented as mean ± SEM. Statistical significance was determined by one-way ANOVA with Tukey's post-hoc test for multiple comparisons. ∗P < 0.05, ∗∗P < 0.01, ∗∗∗P < 0.001, ∗∗∗∗P < 0.0001.

To investigate the potential paracrine effect of chondrocyte-derived RSPO3 on macrophages, we treated BMDMs with recombinant RSPO3 protein (Fig. 5A), and found that RSPO3 potently promoted M2 polarization. Immunofluorescence and flow cytometric analysis demonstrated that RSPO3 significantly attenuated LPS/IFN-γ-induced M1 polarization (marked by reduced CD86) and concurrently promoted a shift towards the M2 phenotype (marked by increased CD163 and CD206) (Fig. 5B and C). WB further confirmed that RSPO3 upregulated key M2 markers (ARG1, CD163) while downregulating the canonical M1 markers (iNOS, CD86) (Fig. 5D). Consistent with this phenotypic switch, RSPO3 treatment changed the cytokines secretion towards an anti-inflammatory state by enhancing the secretion of IL-10 and suppressing that of IL-1β (Fig. 5E). However, a central question was whether RSPO3 influenced macrophages directly or indirectly via other chondrocyte factors. Using indirect co-culture experiments of macrophages and chondrocytes (Fig. 5F), we found that conditioned medium from RSPO3-treated chondrocytes potently induced M2 polarization. But this effect was abolished when Rosmantuzumab was added to the macrophage medium (Fig. 5G). These data establish that RSPO3 functions as a necessary and direct paracrine signal from chondrocytes to macrophages for inducing M2 polarization.

#### 3.4.2 RSPO3 activates the canonical Wnt/β-catenin pathway in macrophages via LGR4/LRP6

**Fig. 6:**
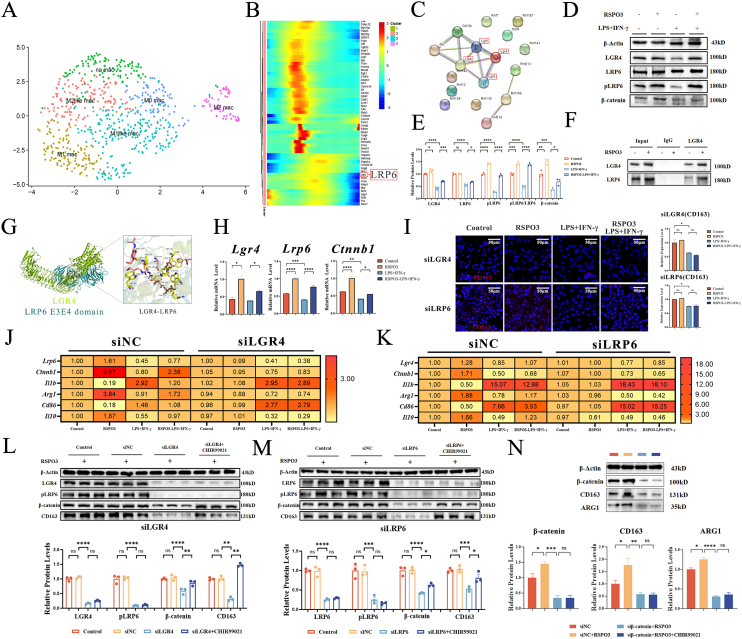
RSPO3 drives M2 polarization through the LGR4/LRP6/β-catenin signaling axis. **A- C.Bioinformatic analysis implicates the function of Wnt/β-catenin pathway in macrophage transformation. (A).**t-SNE visualization of single-cell RNA-seq data (GSE172500) reveals macrophage heterogeneity in OA joints. **(B).**Pseudo-temporal trajectory analysis reconstructing the dynamic gene expression pattern in M2-like macrophages accompanied with the progression of OA. (**C).**Protein interaction network analysis identifies LRP6 as a central hub with high connectivity to LGR4, suggesting their functional cooperation in the Wnt/β-catenin pathway. **D-G.RSPO3 activates the LGR4/LRP6 receptor complex. (D, E).**Western blot images and corresponding quantification show that RSPO3 (40 ng/mL) upregulates the expression levels of LGR4, LRP6, phospho-LRP6 (p-LRP6), and β-catenin in macrophages (n = 3). **(F).**Co-IP analysis confirms that RSPO3 enhances the endogenous LGR4-LRP6 interaction (n = 3). **(G).**Molecular docking model predicts a high-affinity binding interface between LGR4 and the LRP6 E3E4 domain. **H-K.The LGR4/LRP6 complex is essential for RSPO3-induced M2 programming. (H).**qPCR analysis validates RSPO3-induced, increased transcriptional levels of *Lgr4*, *Lrp6*, and *Ctnnb1* (β-catenin) (n = 6). **(I).**Immunofluorescence images show that knockdown of *Lgr4* or *Lrp6* abolishes RSPO3-induced CD163 (M2 marker) expression (scale bar: 50 μm; n = 3). **(J, K).**qPCR analysis of M2 (Arg*1*, *Il10*) and M1 (*Nos2, Il1b*) markers confirms the necessity of LGR4 and LRP6 for RSPO3-mediated M2 reprogramming (n = 6). **L-N.Genetic evidence places β-catenin as the indispensable downstream effector of LGR4/LRP6. (L, M).**Western blot analysis demonstrates that *Lgr4* or *Lrp6* knockdown blocks RSPO3-induced high expression levels of p-LRP6, nuclear β-catenin and CD163. Crucially, the β-catenin agonist CHIR99021 (3 μM) rescues the inhibited M2 polarization, proving β-catenin acts downstream. **(N).**Conversely, in *Ctnnb1*(β-catenin)-knockdown macrophages, RSPO3 fails to induce the expression of CD163, and CHIR99021 cannot restore M2 polarization (n = 3). Data are mean ± SEM. Statistics: one-way ANOVA with Tukey's post-hoc test. ∗P < 0.05, ∗∗P < 0.01, ∗∗∗P < 0.001, ∗∗∗∗P < 0.0001.

We next investigated the mechanism by which RSPO3 signals in macrophages. As RSPO3 is a known ligand for LGR family receptors (particularly LGR4) that canonically activate the Wnt/β-catenin pathway [48], we hypothesized a similar pathway in macrophages (Fig. S4). To identify the relevant co-receptors in an OA context, we analyzed a single-cell RNA-seq dataset of synovial macrophages in OA models (GSE172500) [49], which revealed significant heterogeneity and dynamic gene expression patterns among different macrophage subtypes. Then, we noticed the significantly down-regulated expression of low-density lipoprotein receptor-related protein 6 (LRP6) in vivo accompanied with the progress of OA (Fig. 6A and B). A protein-protein interaction (PPI) network analysis focused on the Wnt/β-catenin pathway identified LRP6 as a core component with high potential for interaction with LGR4 (Fig. 6C). We next experimentally validated this bioinformatic prediction. WB analysis confirmed that RSPO3 treatment not only increased the expression levels of LGR4 but also induced phosphorylation of LRP6 (p-LRP6), the essential activation step for the canonical Wnt pathway (Fig. 6D and E). Co-immunoprecipitation (Co-IP) assays confirmed that RSPO3 enhanced the physical interaction between LGR4 and LRP6 (Fig. 6F). A molecular docking model provided structural insights, predicting a high-affinity binding interface between LGR4 and the LRP6 E3E4 domain, stabilized by key interactions such as a salt bridge between ASP-735 (LGR4) and ARG-675/688 (LRP6) (Fig. 6G). This receptor complex activation led to the stabilization and nuclear translocation of β-catenin, the central transcriptional effector of the canonical Wnt pathway (Fig. 6D–H).

#### 3.4.3 The LGR4/LRP6/β-catenin axis is necessary for RSPO3-induced M2 polarization

To establish a causal link between Wnt/β-catenin pathway activation and the M2 phenotype, we performed loss-of-function experiments (Fig. S5). siRNA-mediated knockdown of either *Lgr4* or *Lrp6* effectively abolished RSPO3-induced M2 polarization, as evidenced by immunofluorescence images and the expression of M2/M1 markers (Fig. 6I–M). This genetic interruption also prevented RSPO3-induced LRP6 phosphorylation, β-catenin activation, and IL-10 secretion (Fig. 6J–M). We then asked whether β-catenin activation was the essential downstream event. Treatment with CHIR99021, a specific and potent GSK-3β inhibitor that stabilizes β-catenin [50], fully rescued the M2 polarization program in *Lgr4-*or *Lrp6-*knockdown macrophages (Fig. 6L and M). To provide direct genetic evidence, we utilized a macrophage-specific β-catenin knockdown model to further support the conclusions. RSPO3 failed to induce M2 polarization in β-catenin-knockdown macrophages, whereas it effectively did so in negative controls. Crucially, CHIR99021 was unable to rescue the M2 polarization effect in β-catenin-knockdown macrophages (Fig. 6N), which provides strong evidence that the rescue effect of CHIR99021 is entirely dependent on the presence of β-catenin. Thereby, these data specifically confine the downstream mechanism for RSPO3's pro-M2 effect to β-catenin.

In summary, these data delineate a complete and definitive signaling axis. RSPO3 directly binds to and activates the LGR4/LRP6 receptor complex on macrophages, leading to β-catenin stabilization and nuclear translocation, which is necessary for inducing the pro-repair M2 phenotype.

### 3.5 RSPO3 is both sufficient and necessary for the therapeutic efficacy of NsPEFs-ADSCs-EVs in OA

#### 3.5.1 Exogenous RSPO3 is sufficient to achieve the dual therapeutic effects of NsPEFs-ADSCs-EVs

**Fig. 7:**
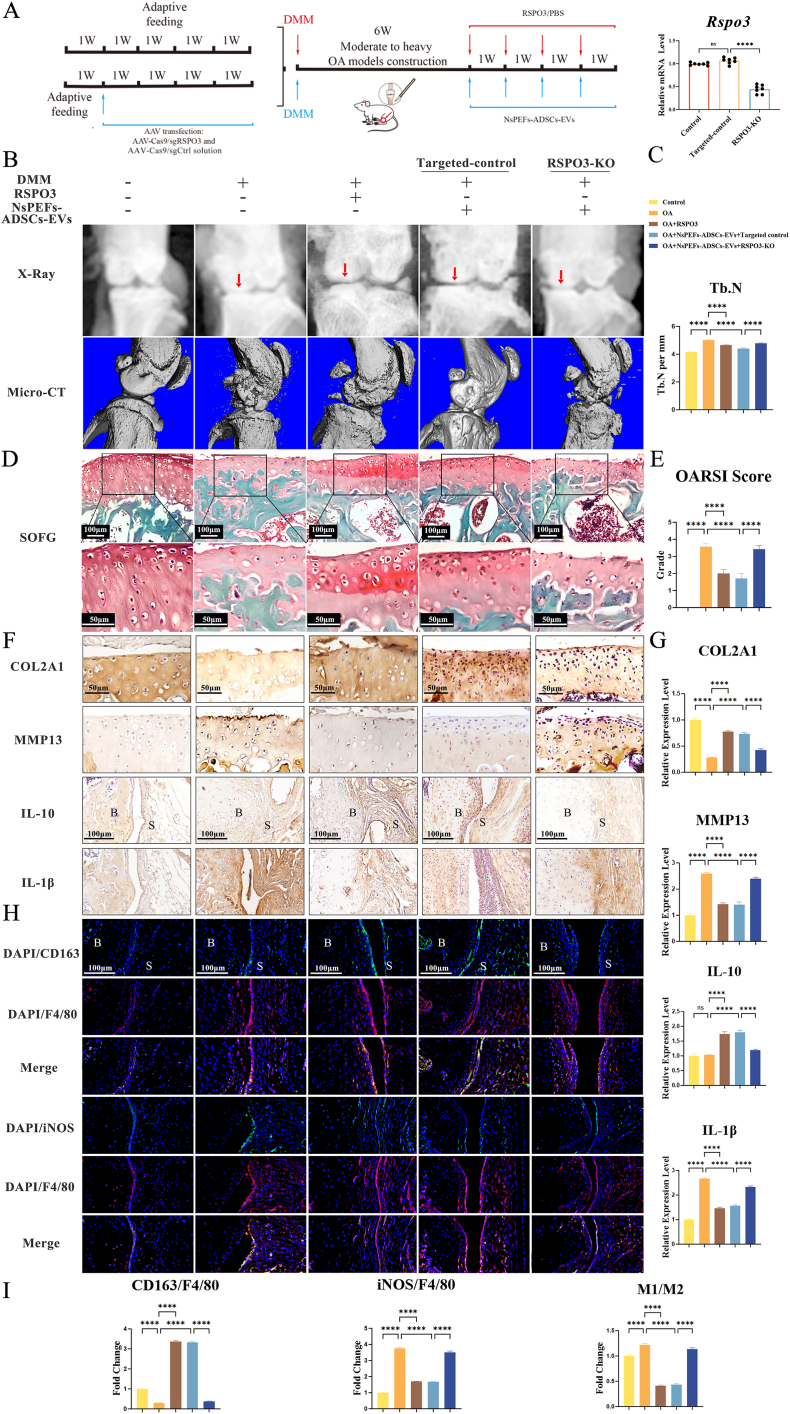
RSPO3 is both sufficient and necessary for the therapeutic effects of NsPEFs-ADSCs-EVs in OA **A**.Left: Experimental timeline illustrating the establishment of the DMM-induced OA model and a local RSPO3-knockout (RSPO3-KO) milieu. Subsequent therapeutic interventions were carried out with either recombinant RSPO3 protein or NsPEFs-ADSCs-EVs. Right: qPCR analysis demonstrates the efficient knockdown of RSPO3 in cartilage tissue following AAV-sgRSPO3 injection (n = 7 mice per group). **B.**Representative X-ray images (arrows highlighting medial joint space) and sagittal reconstruction images of knee joints using micro-CT from the indicated experimental groups. **C.**Quantification of trabecular number (Tb.N) from micro-CT analysis (n = 7). **D, E.**Representative SOFG staining of knee joint sections (scale bars: 100 μm and 50 μm) and corresponding quantitative OARSI scoring (n = 7). **F, G.** Left: Immunohistochemical (IHC) staining for anabolic (COL2A1) and catabolic (MMP13) markers in cartilage, and for anti-inflammatory (IL-10) and pro-inflammatory (IL-1β) cytokines in synovium (scale bar: 50 μm and 100 μm). Right: Quantitative analysis of IHC staining intensity (n = 7). **H, I.** Top: Representative immunofluorescence images of synovial tissue co-stained for the pan-macrophage marker F4/80 (red) with the M2 marker CD163 (green) or the M1 marker iNOS (green). Nuclei are stained with DAPI (blue) (scale bar: 100 μm). Bottom: Quantification of the fluorescence intensity ratio of CD163/F4/80 and iNOS/F4/80 (n = 7). Data are mean ± SEM. Statistics: one-way ANOVA with Tukey’ s post-hoc test. ∗P < 0.05, ∗∗P < 0.01, ∗∗∗P < 0.001, ∗∗∗∗P < 0.0001.

To determine if RSPO3 alone could exhibit the dual therapeutic benefits, we performed gain-of-function experiments in the DMM-induced OA model. Mice received weekly intra-articular injections of recombinant RSPO3 protein (40 ng/mL in 10 μL PBS, a dose extrapolated from in vitro efficacy, previous studies and initial in-vivo dose exploration experiments; Fig. S6) or PBS control, starting six weeks post-surgery (Fig. 7A, upper panel). Micro-CT and radiographic analysis at 10 weeks post-surgery revealed that RSPO3 treatment significantly mitigated OA-associated joint space narrowing and subchondral bone sclerosis compared to the OA group (Fig. 7B and C). Histologically, Safranin O/Fast Green (SOFG) staining demonstrated significant preservation of articular cartilage architecture and proteoglycan content in RSPO3-treated joints, leading to significantly lower OARSI scores (Fig. 7D and E). Immunohistochemistry confirmed the pro-anabolic shift in cartilage compared to the OA group, with increased collagen type II (COL2) and decreased MMP13 expression (Fig. 7F and G).

Crucially, RSPO3 treatment also exerted the immunomodulatory effects. Immunofluorescence analysis of synovial tissue showed a significant increase in the ratio of M2 (CD163^+^) to M1 (iNOS^+^) macrophages and a consistent shift in the cytokine balance towards an anti-inflammatory state, with elevated IL-10 and suppressed IL-1β levels (Fig. 7F–I). Collectively, these data establish that exogenous RSPO3 is sufficient to achieve the dual pro-chondrogenic and pro-M2 properties of NsPEFs-ADSCs-EVs.

#### 3.5.2 The therapeutic efficacy of NsPEFs-ADSCs-EVs is dependent on host-derived RSPO3

We next investigated whether the therapeutic effects of NsPEFs-ADSCs-EVs were dependent on the induction of endogenous RSPO3 in the joint. We employed an intra-articular injection of AAV-Cas9/sgRSPO3 to create a joint-specific RSPO3-deficient (RSPO3-KO) microenvironment prior to NsPEFs-ADSCs-EVs treatment (Fig. 7A, lower panel).

Strikingly, the knockout of local RSPO3 expression abrogated the therapeutic benefits of NsPEFs-ADSCs-EVs. In RSPO3-KO mice, NsPEFs-ADSCs-EVs failed to improve joint architecture, cartilage histology (OARSI scores), or the COL2/MMP13 balance, outcomes that were significantly improved by NsPEFs-ADSCs-EVs in targeted-control mice (Fig. 7B–G). Similarly, the ability of NsPEFs-ADSCs-EVs to promote synovial M2 macrophage polarization and shift the cytokine profile towards anti-inflammation was significantly declined in the absence of host RSPO3 (Fig. 7F–I).

In summary, these gain-of-function and loss-of-function experiments in vivo establish RSPO3 as the central effector molecule that is both sufficient and necessary for the dual therapeutic effects of NsPEFs-ADSCs-EVs in OA.

### 3.6 NsPEFs-ADSCs-EVs induce chondrocyte RSPO3 secretion via an ITGA4/PI3K/Akt-dependent mechanism

**Fig. 8:**
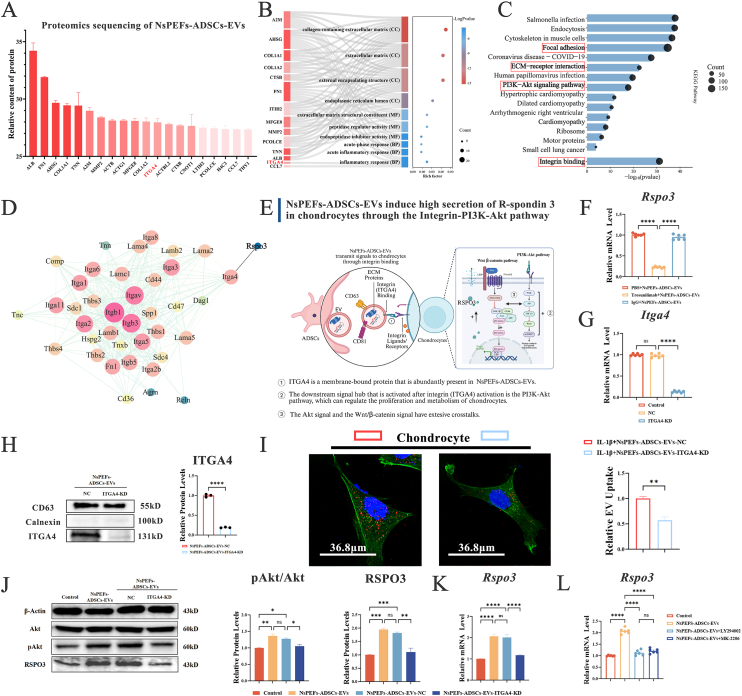
NsPEFs-ADSCs-EVs induce RSPO3 secretion via an ITGA4/PI3K/Akt-dependent mechanism. **A- D.Proteomic profiling identifies ITGA4 as a key mediator linking NsPEFs-ADSCs-EVs to RSPO3. (A).**Significantly enriched proteins in NsPEFs-ADSCs-EVs using proteomic analysis (n = 3). **(B, C).**Gene Ontology and KEGG pathway enrichment analyses of proteins in NsPEFs-ADSCs-EVs, highlight integrin binding, cell adhesion and PI3K-Akt signaling. **(D).**The protein-protein interaction network integrating RSPO3 with top enriched EV proteins, reveals a potential functional link with ITGA4. **E- I.EV-surface ITGA4 is essential for chondrocyte targeting and RSPO3 induction. (E).**The schematic hypothesizes the ITGA4-initiated signaling axis related to RSPO3 secretion. **(F)** qPCR analysis shows that the increased transcriptional level of *Rspo3* in chondrocytes treated with NsPEFs-ADSCs-EVs is inhibited by an ITGA4-neutralizing antibody (Trosunilimab) (n = 6). **(G).**qPCR validation of *Itga4* knockdown efficiency in ADSCs (n = 6). **(H).**Western blot analysis confirms the successful generation of ITGA4-deficient EVs (NsPEFs-EVs-ITGA4-KD) from *Itga4*-knockdown ADSCs, while maintaining EV purity (CD63^+^/Calnexin^−^) (n = 3). **(I)** Cellular uptake of DiR-labeled NsPEFs-ADSCs-EVs-ITGA4-KD by chondrocyte is significantly impaired compared to that of NsPEFs-ADSCs-EVs-NC. Quantification of fluorescence intensity is shown (scale bar: 36.8 μm; n = 3). **J-L.ITGA4 initiates RSPO3 expression through the PI3K/Akt pathway. (J).**Western blot analysis of Akt phosphorylation (p-Akt) and RSPO3 in chondrocytes treated with the indicated EVs (n = 3). **(K).**qPCR analysis of *Rspo3* confirms that ITGA4-deficient EVs fail to induce RSPO3 expression (n = 6). **(L).**Pharmacological inhibition of PI3K (LY294002) or Akt (MK-2206) abolishes NsPEFs-ADSCs-EVs-induced *Rspo3* upregulation in chondrocytes (n = 6). Data are presented as mean ± SEM. Statistical significance was determined by unpaired two-tailed Student's t-test or one-way ANOVA with Tukey's post-hoc test. ∗P < 0.05, ∗∗P < 0.01, ∗∗∗P < 0.001, and ∗∗∗∗P < 0.0001.

To decipher the initiating signal from NsPEFs-ADSCs-EVs, we analyzed their protein cargo. Proteomic analysis identified numerous enriched proteins, among which the membrane-bound integrin subunit α4 (ITGA4) was a candidate of functional signals due to its established role in cell-cell communication and signaling initiation [51] (Fig. 8A and B). Pathway analysis of EV proteins highlighted enrichment in “ECM-receptor interaction” and “Focal adhesion” pathways, consistent with a role in integrin-mediated signaling (Fig. 8C). Bioinformatic analysis hints that ITGA4 is inter-related with RSPO3 (Fig. 8D). Given that integrin activation often converges on the PI3K-Akt pathway [52], which exhibits extensive crosstalk with Wnt/β-catenin signaling [53], we proposed that EV-surface ITGA4 might bind to chondrocyte surface receptors to initiate intracellular signals and culminate in RSPO3 production (Fig. 8E).

We then tested the functional necessity of EV-borne ITGA4. Pre-incubation of NsPEFs-ADSCs-EVs with a function-blocking anti-ITGA4 antibody significantly attenuated their ability to upregulate *RSPO3* transcription in chondrocytes (Fig. 8F). To definitively establish the necessity of EV-borne ITGA4 and investigate the immediate downstream signaling events, we genetically silenced *Itga4* in ADSCs prior to EVs collection (Fig. 8G). Western blot analysis confirmed the successful generation of ITGA4-knockdown EVs (NsPEFs-ADSCs-EVs-ITGA4-KD) (Fig. 8H). The cell uptake experiment confirmed that the ability of chondrocytes to uptake NsPEFs-ADSCs-EVs-ITGA4-KD significantly decreased, suggesting that ITGA4 might be involved in the chondrocyte-targeting and endocytosis activation (Fig. 8I). The results also indicated that NsPEFs-ADSCs-EVs induced phosphorylation of Akt (Ser473) and stimulated RSPO3 expression. Reversely, the activation was abolished in chondrocytes treated with ITGA4-deficient NsPEFs-ADSCs-EVs (Fig. 8J and K). Pharmacological inhibition of PI3K (LY294002 [54]; 50 μM) or Akt (MK-2206 [55]; 10 μmol/L) significantly suppressed the ability of NsPEFs-ADSCs-EVs to up-regulate the expression of *Rspo3* (Fig. 8L).

Collectively, these data establish the ITGA4/PI3K/Akt axis as the critical upstream mechanism through which NsPEFs-ADSCs-EVs instruct chondrocytes to become a source of therapeutic RSPO3.

## 4 Discussion

Our study elucidates a previously unrecognized mechanism axis that synchronously coordinates cartilage repair and immune resolution in OA, orchestrated by the secretory factor RSPO3. We demonstrate that NsPEFs-engineered ADSCs-EVs, acting as a precision delivery system, instruct chondrocytes to assume a novel immunomodulatory role via the ITGA4-initiated secretion of RSPO3. This chondrocyte-derived RSPO3, in turn, executes a dual-function: promoting anabolic activity in cartilage and driving the phenotypic conversion in synovial macrophages towards the pro-repair M2 state by activating the LGR4/LRP6/β-catenin pathway. This "one stone, two birds" mechanism positions RSPO3 as a central mediator and a compelling therapeutic target for OA. The present work not only provides a comprehensive mechanistic framework from EV engineering to in vivo efficacy but also addresses key translational considerations.

### 4.1 Methodological innovation and conceptual shift in chondrocyte biology

A pivotal contribution of our work is the synergistic advancement of both technical and conceptual frontiers. Technically, we addressed a critical translational bottleneck in EV therapy by employing a non-invasive, non-genetic NsPEFs preconditioning platform to generate ADSCs-EVs with superior yield and bioactivity. Conceptually, our findings propose a paradigm shift in understanding chondrocyte function within the OA joint. Traditionally viewed as passive victims of inflammatory assault, we unveil chondrocytes as active architects of the immune microenvironment. Upon receiving specific cues from NsPEFs-ADSCs-EVs, they are transformed into potent signaling hubs that, through RSPO3 secretion, directly promote the construction of a tissue-reparative milieu. This aligns with the emerging recognition of stromal cells as key immunomodulators in tissue homeostasis [56], but assigns a specific molecular, RSPO3, to this function in chondrocytes.

### 4.2 RSPO3 as a dual-function effector synchronizing tissue repair and immune resolution

#### 4.2.1 RSPO3 enhances cartilage anabolism via an autocrine signaling pathway

This study demonstrates that RSPO3 secreted by chondrocytes can directly regulate chondrocyte proliferation and matrix synthesis in an autocrine manner. The specific signaling mechanism was not further investigated in the present study. But, as a potent agonist of both canonical and non-canonical Wnt pathways, RSPO3 likely exerts its cartilage-repairing effect through activation of Wnt signaling. Such activation may occur in an LGR-dependent manner [57] or be mediated by other receptors, such as membrane-bound heparin sulfate proteoglycans (HSPGs) [58]. Current evidence indicates that the canonical Wnt/β-catenin pathway does not serve as the principal signaling mechanism driving cartilage self-renewal and repair. On the contrary, it may suppress the synthesis of collagen type II and promote chondrocyte hypertrophy or ossification [59,60]. Interestingly, activation of the non-canonical Wnt pathway (Wnt/PCP signaling) contributes to the maintenance of cartilage morphological structure [61]. Therefore, we propose that the direct pro-chondrogenic property by RSPO3 may depend on HSPG-mediated signal reception and subsequent activation of Wnt/PCP signaling. Future work will focus on further mechanistic validation to strengthen these findings.

#### 4.2.2 RSPO3 as a novel immunometabolic mediator in OA

The immunomodulatory role of RSPO3 uncovered here constitutes a significant expansion of its biological function. While its functions in development, angiogenesis and cancer are well-documented [21,62], its capacity to function as a chondrocyte-derived signal for M2 macrophage polarization is previously unreported. We mechanistically pinpoint that RSPO3 achieves this function by acting on the LGR4/LRP6 co-receptor complex in macrophages, thereby activating the canonical Wnt/β-catenin pathway. This discovery effectively bridges the fields of Wnt developmental biology and joint immunometabolism, nominating RSPO3 as a novel local immunometabolic mediator that promotes the resolution of inflammation, potentially circumventing the systemic side effects associated with broad immunosuppressants [63,64].

#### 4.2.3 The unique function of RSPO3 in the complex RSPO family

The RSPO family of secreted proteins exhibit complex and often opposing roles in joint homeostasis. Notably, RSPO2 exacerbates cartilage catabolism, and its inhibition attenuates OA progression in mice [65,66]. Conversely, RSPO1 demonstrates chondroprotective effects in arthritis models [67], suggesting that each individual RSPO protein has a unique activity. We propose several explanations for the unique beneficial role of RSPO3 uncovered in our study. Firstly, while all RSPOs enhance Wnt signaling, they exhibit preferential binding to different receptors. The expression patterns of these receptors on distinct cell types within the joint can influence the biological outcome. Our data unequivocally show that in the context of the OA joint, the RSPO3-LGR4/LRP6 axis on macrophages drives a potent pro-repair M2 polarization program, an effect not reported for RSPO2. This suggests that the immunomodulatory function we described may be a unique property of the RSPO3-LGR4 interaction in the specific pathological setting and target cells. Secondly, the opposite expression patterns of RSPO2 and RSPO3 in synovial fibroblasts in arthritis models further provide indirect evidence of their distinct functional roles [68], which also explain why RSPO3 was discovered in the reparative mechanism while RSPO2 was discovered in the pathological mechanism. Thirdly, emerging evidence indicates that R-spondins can signal through pathways beyond canonical Wnt/β-catenin. Given the inherent antagonism between canonical and non-canonical Wnt signaling pathways [69] and the complexity of their regulatory networks, the functions of RSPOs should not be oversimplified.

### 4.3 ITGA4: from a surface marker to a functional determinant

The finding that EV-borne ITGA4 is indispensable for inducing RSPO3 expression in chondrocytes provides a rational basis for the observed superiority of NsPEFs-ADSCs-EVs. We further substantiated the mechanism that ITGA4-initiated PI3K-Akt signaling likely serves as the proximate trigger for RSPO3 up-regulation. This finding transcends the conventional understanding of ITGA4 as a simple homing receptor [51]. It demonstrates that EV-enriched ITGA4 not only mediates the specific anchoring of NsPEFs-ADSCs-EVs but also directly triggers an intracellular functional signaling cascade (PI3K-Akt). This finding further demonstrates that NsPEFs treatment does not simply increase EVs production but functionally remodels the membrane protein composition and signaling activation capacity of EVs.

Although this study has identified the core role of EV-ITGA4, the interacting receptors on chondrocytes remain an open question that needs to be clarified. VCAM-1 is the most classic and clearly characterized receptor of ITGA4 [70] (Fig. S7A). The clinical gene database shows that VCAM-1 is significantly upregulated in chondrocytes in the inflammatory OA environment [71,72] (Fig. S7B). The current understanding is that the up-regulation of VCAM-1 in the cartilage layers of osteoarthritis patients is believed to promote immune cell adhesion and damage [73]. However, considering that the ability of chondrocytes to uptake NsPEFs-ADSCs-EVs is also significantly enhanced in an inflammatory environment, we speculate that when VCAM-1 is captured by NsPEFs-ADSCs-EVs rather than immune cells, it may also be related to the transmission of pro-repair and anti-inflammatory signals. The preliminary WB and qPCR results showed that the VCAM-1 neutralizing antibody could significantly inhibit the expression of RSPO3 induced by NsPEFs-ADSCs-EVs (Fig. S7C and D), which strongly suggests that VCAM-1 is a crucial receptor on chondrocytes for initiating the pro-repair and anti-inflammatory mechanisms. Nevertheless, the precise nature of this binding interaction warrants further validation in subsequent studies.

Collectively, this study not only establishes the core function of ITGA4 in the NsPEFs-ADSCs-EVs therapy, but also provides a new paradigm for the rational engineering design of EVs. ITGA4 has been confirmed as a "effector-target" dual-functional molecule, which provides a clear direction for the future design of a new generation of therapeutic EVs. It can be attempted to over-express or display ITGA4 on the membrane through genetic engineering, in order to actively enhance the chondrocyte-targeting and repair-initiating capabilities of EVs. The NsPEFs technology plays a dual role in this process. It is not only a preparation method for efficiently enriching functional membrane proteins (such as ITGA4), but also verifies the feasibility of directing the modification of EV surface properties through physical intervention to give them new functions. In the future, by drawing on this idea, a series of engineered EVs expressing different target-signal integrins can be constructed for precise treatment of different target cells or pathological conditions, thereby promoting the development of EV therapy towards the next generation of designable and programmable direction.

### 4.4 Translational implications, limitations and future directions

From a translational standpoint, our work proposes three promising, mutually reinforcing strategies. First, given the human transcriptomic data presented (E-MTAB-4304), recombinant human RSPO3 protein itself may emerge as a novel, locally-applied biologic agent capable of simultaneously inhibiting cartilage degradation and resolving synovitis for human OA, especially for the OA population with significantly lower expression of RSPO3. Second, the NsPEFs presents a viable and efficient path for the large-scale manufacturing of high-potency EVs, moving the EV field closer to clinical application. Third, the targeting and functional properties of ITGA4 make it a potential engineering site, which is expected to facilitate the targeted cartilage repair using nanomaterials or EVs. Furthermore, the potential of these strategies extends beyond the knee. Given the critical role of inflammation and cartilage degradation in temporomandibular joint OA (TMJOA), a condition with high clinical need and limited options [74,75], our therapeutic strategies represent promising avenues for future investigation in small joint pathologies.

Although we have strongly demonstrated the crucial roles of RSPO3 and ITGA4 in the NsPEFs-ADSCs-EVs treatment of OA, these findings do not mean that RSPO3 and ITGA4 are the sole effective substances in the therapeutic process. Other active substances in NsPEFs-ADSCs-EVs may be involved in various aspects and stages of the treatment. Therefore, a comprehensive understanding of the functional properties of NsPEFs-ADSCs-EVs requires more complex and in-depth research in the future. Furthermore, this study employed a young male mouse model of DMM. The reasons for choosing this model are as follows. Firstly, this model is widely used in the field. Secondly, controlling factors related to OA (such as age, gender, metabolism) help to minimize individual differences and reduce interference. Taking into account the relatively low theoretical generalizability of a single model, future studies need to verify the generalizability of this therapy in aging or metabolic OA models and in female animals. Advanced preclinical models will be also necessary. Finally, although we propose that VCAM-1 may act as the receptor of ITGA4 to promote the activation of the PI3K/Akt pathway and the high expression of RSPO3, the verification of the necessity of this receptor function is not sufficient. In the future, we will conduct more detailed and comprehensive verification to determine whether VCAM-1 or other adhesion molecules are involved in this specific event between EVs and chondrocytes, in order to make our research mechanism more complete and systematic.

## 5 Conclusion

In this study, we discovered that the engineered ADSCs-EVs by NsPEFs can drive chondrocytes to efficiently secrete RSPO3 through the ITGA4-initiated signaling axis. RSPO3 then plays a dual function. On one hand, it activates the anabolic program within chondrocytes through autocrine mode. On the other hand, it induces macrophages to polarize towards the M2 anti-inflammatory phenotype through the LGR4/LRP6/β-catenin pathway. Collectively, this study not only reveals that RSPO3 is a crucial new target in joint homeostasis, but also provides NsPEFs as an engineering platform for scalable production of efficient EVs. Our work has paved the way for developing the next generation of OA therapies aimed at the whole joint improvement.

## CRediT authorship contribution statement

**Yushan Wang:** Writing – original draft. **Yingjie Gao:** Project administration. **Zhiyan Cao:** Writing – review & editing. **Mingjie Dong:** Conceptualization. **Pengfei Shao:** Data curation. **Hao Fan:** Investigation. **Zijian Guo:** Data curation. **Xiaoyong Hu:** Writing – review & editing. **Wenxiang Cheng:** Resources. **Pengcui Li:** Supervision. **Wei Zhang:** Methodology. **Yi Feng:** Conceptualization. **Panfeng Fu:** Software. **Zigang Ge:** Resources. **Jiake Xu:** Writing – review & editing. **Chuan Xiang:** Project administration, Funding acquisition.

## Ethics approval and consent to participate

The protocols have been reviewed and approved by the Ethics Committee of the Second Hospital of Shanxi Medical University (DW2025011) and the Institutional Animal Care and Use Committee (2019-0004) in Shanxi Medical University.

## Availability of data and materials

The source data utilized in this study can be obtained upon reasonable request by contacting the corresponding author.

## Funding

This study was supported by grants from the National Key R&D Program of China (2024YFA0919200), Shenzhen Medical Research Fund (B2302005), the Postgraduate Education Innovation program of Shanxi Province (2024SJ155), the central government guiding local funds for scientific and technological development (YDZJSX20231A062), and scientific and technological achievements transformation guidance project of Shanxi Province (202204021301067). This study was also supported in part by the 10.13039/501100001809National Natural Science Foundation of China (82350710800, 82374470), Shenzhen Science and Technology Program (KCXFZ20240903094059020, SGDX20240115112400001), and Key Field Special Project of Guangdong Provincial Colleges and Universities (2024ZDZX2014).

## Declaration of competing interest

The authors hereby affirm that they have no financial, professional, or personal conflicts of interest that could influence or bias the research, findings, or conclusions presented in this study.

## List of abbreviations

**OA**: Osteoarthritis
**ADSCs-EVs**: Adipose derived stem cells-extracellular vesicles
**RSPO3**: Rspondin 3
**LGR4**: Leucine-rich repeat-containing G-protein coupled receptor 4
**LRP6**: Low-density lipoprotein receptor-related protein 6
**NsPEFs**: Nanosecond pulsed electric fields
**NTA**: Nanoparticle tracking analysis
**WB**: Western blotting
**BMDMs**: Bone marrow derived macrophages
**DMM**: Destabilization of the medial meniscus
**SOFG**: Safarnin O - Fast Green staining
**pLRP6**: phosphorylation of LRP6
**GO**: Gene Ontology
**KEGG**: Kyoto Encyclopedia of Genes and Genomes
**PPI**: Protein-protein interaction
**Co-IP**: Co-immunecipation
ITGA4: Integrin subunit alpha 4
VCAM-1: Vascular cell adhesion molecule 1

## Footnotes

2 ITGA4: integrin subunit alpha 4.

3 RSPO3: R-spondin 3.

4 LGR4: Leucine-rich repeat-containing G-protein coupled receptor 4.

5 LRP6:Low-density lipoprotein receptor-related protein 6.
