# Supplementary material for: NsPEFs-enriched ADSCs-EVs alleviate osteoarthritis via RSPO3-mediated dual pro-chondrogenic and pro-M2 macrophage properties
Source: Bioact Mater. 2026 Jan 17;59:763–80. doi: 10.1016/j.bioactmat.2026.01.006 (PMC12856188; doi:10.1016/j.bioactmat.2026.01.006)
Supplement: Multimedia component 1 [file mmc1.docx]

Supplementary Materials

**
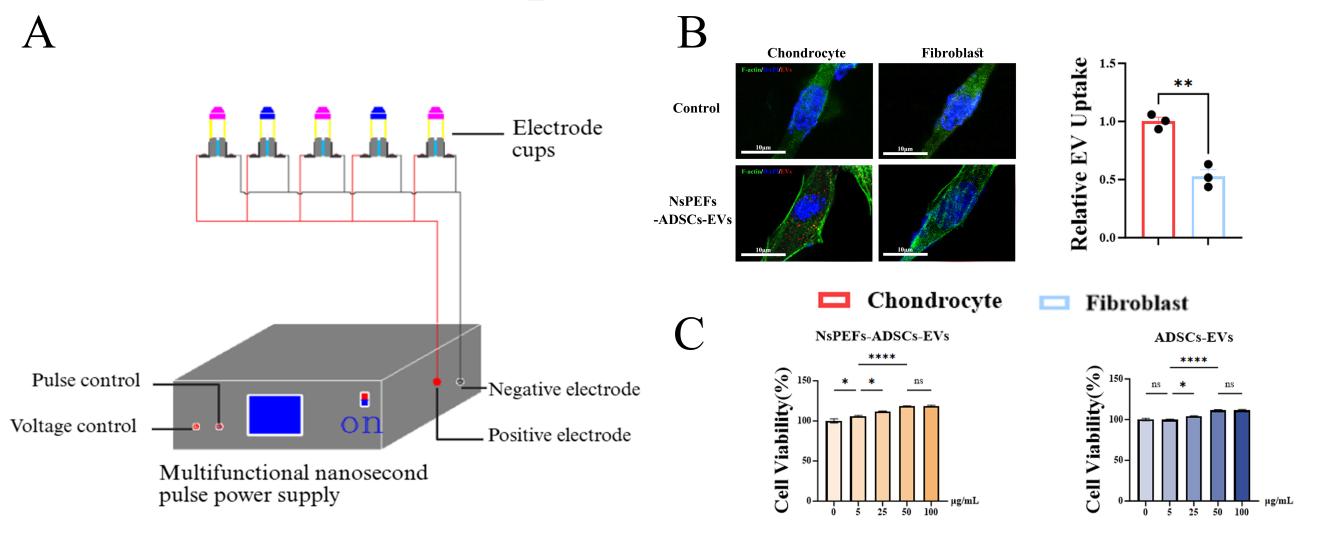
**

**Fig. S1. NsPEFs-ADSCs-EVs are more significantly taken up by chondrocytes compared to synovial fibroblasts, thereby promoting chondrocytes proliferation in a dose-dependent manner.**

**A.**Schematic diagram of the intervention process ADSCs suspension by the NsPEFs device. **B.**Cellular uptake of DiR-labeled NsPEFs-ADSCs-EVs (red) by chondrocytes and synovium fibroblasts in co-culture system, with nuclei stained by DAPI (blue). Quantification of fluorescence intensity is shown (scale bar: 10 μm; n=3). **C.**CCK8 assay of chondrocytes treated with different concentrations of NsPEFs-ADSCs-EVs or Ctrl-ADSCs-EVs (n=6). Data are presented as mean ± SEM. Statistical significance was determined by unpaired two-tailed Student’s t-test or one-way ANOVA with Tukey’s post-hoc test. *P < 0.05, **P < 0.01, ***P < 0.001, and ****P < 0.0001; ns: not significant.


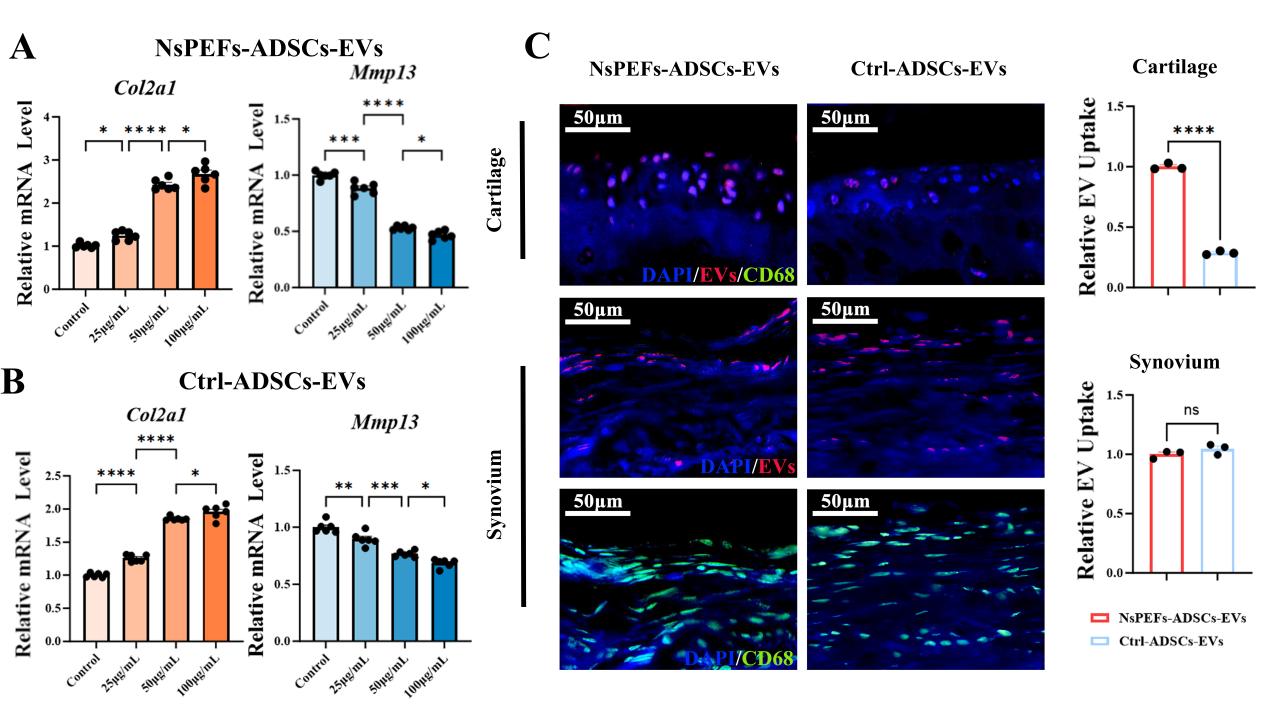


**Fig. S2 NsPEFs-ADSCs-EVs are more efficiently internalized by cartilage and promote the anabolism in a dose-dependent manner.**

**A, B.**qPCR analysis of anabolic (*Col2a1)* and catabolic (*Mmp13*) genes shows that NsPEFs-ADSCs-EVs and Ctrl-ADSCs-EVs promote the matrix homeostasis of cartilage in a dose-dependent manner (n=6). **C.**Immunofluorescence analysis demonstrates the co-localization of EVs with both cartilage layer and synovial tissues (n=3; scale bar: 25 μm). Data are presented as mean ± SEM. Statistical significance was determined by unpaired two-tailed Student’s t-test or one-way ANOVA with Tukey’s post-hoc test. *P < 0.05, **P < 0.01, ***P < 0.001, and ****P < 0.0001; ns: not significant.

**
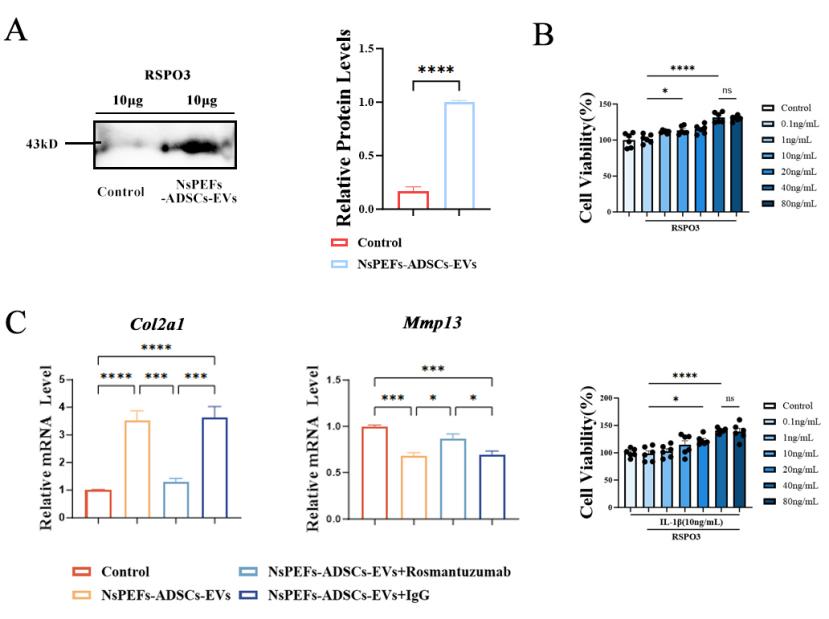
**

**Figure S3. NsPEFs-ADSCs-EVs induce chondrocytes to express secreted RSPO3, which promotes chondrocytes proliferation in a dose-dependent manner.**

**A**.The protein levels of RSPO3 in the culture supernatant before or after the intervention of NsPEFs-ADSCs-EVs (n=3). **B.**The results of CCK8 assay for chondrocytes after intervention with different doses of RSPO3 (n=6). **C.**qPCR analysis demonstrates that the pro-chondrogenic effect of NsPEFs-ADSCs-EVs is influenced by RSPO3 (n=6). Data are presented as mean ± SEM. Statistical significance was determined by unpaired two-tailed Student’s t-test or one-way ANOVA with Tukey’s post-hoc test. *P < 0.05, **P < 0.01, ***P < 0.001, and ****P < 0.0001; ns: not significant.

**
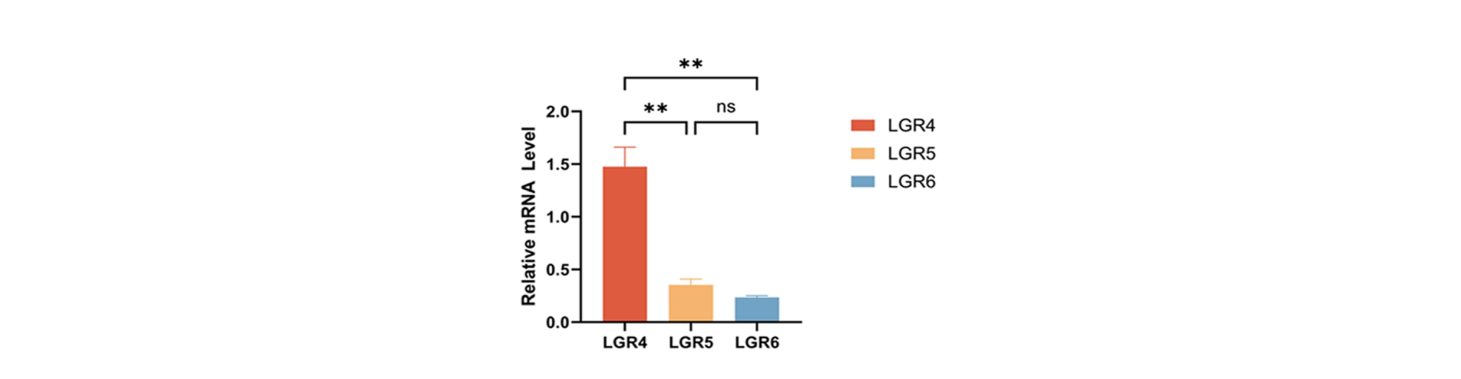
**

**Figure S4. RSPO3 has the highest sensitivity to LGR4 within the LGR family.**

qPCR analysis of *Lgr4, Lgr5 and Lgr6* genes in macrophages after RSPO3 intervention (n=6). Data are presented as mean ± SEM. Statistical significance was determined by one-way ANOVA with Tukey’s post-hoc test for multiple comparisons. *P < 0.05, **P < 0.01, ***P < 0.001, ****P < 0.0001; ns: not significant.

**
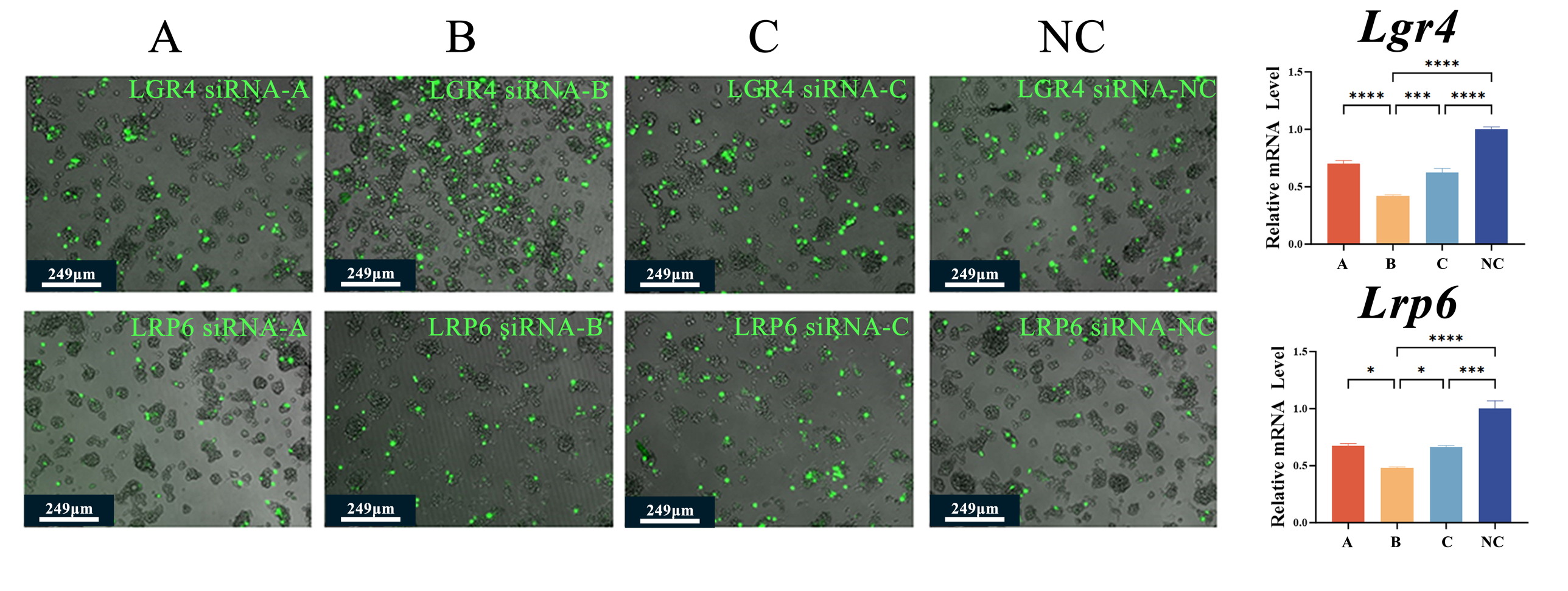
**

**Figure S5. The transfection efficiency of siRNA targeting different sites of *Lgr4* and *Lrp6*.**

Bright-field and fluorescence images showing the transfection efficiency of three different transfection targets for *Lgr4 or Lrp6* (scale bar: 249 μm). qPCR analysis of *Lgr4* and *Lrp6* in macrophages after transfection (n=6). Data are presented as mean ± SEM. Statistical significance was determined by one-way ANOVA with Tukey’s post-hoc test for multiple comparisons. *P < 0.05, **P < 0.01, ***P < 0.001, ****P < 0.0001.

**
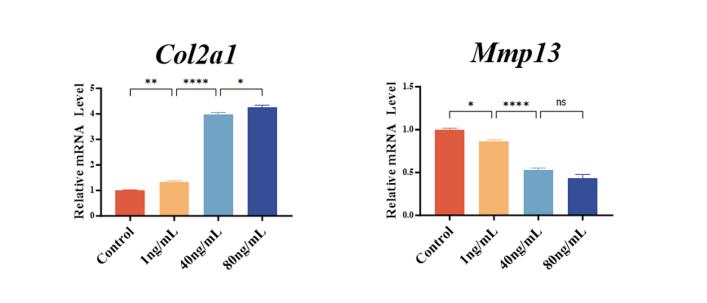
**

**Fig. S6 RSPO3 promotes the anabolism of cartilage in a dose-dependent manner.**

qPCR analysis of anabolic (*Col2a1*) and catabolic (*Mmp13*) genes in cartilage tissues (n=3). Data are presented as mean ± SEM. Statistical significance was determined by one-way ANOVA with Tukey’s post-hoc test for multiple comparisons. *P < 0.05, **P < 0.01, ***P < 0.001, ****P < 0.0001.

**
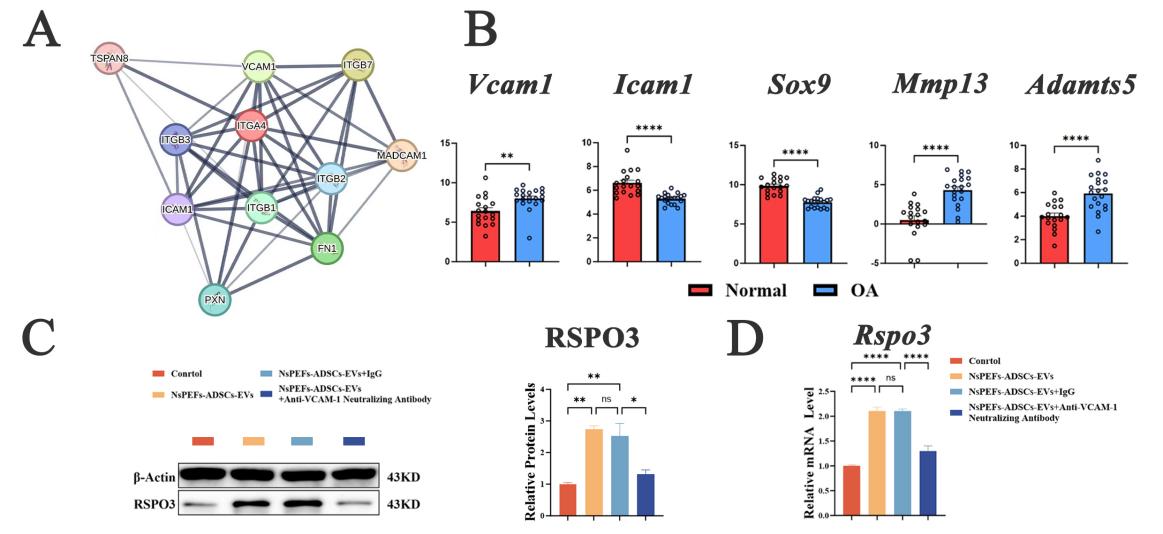
**

**Fig. S7 VCAM-1 is a potential receptor on chondrocytes that receives the signal from EV-surface ITGA4.**

**A.**PPI network analysis predicts VCAM-1 as a classical receptor for ITGA4. **B.**Re-analysis of a human transcriptomic dataset (GSE114007) confirms the significant up-regulation of *Vcam1* gene in OA cartilage (n=18 for normal group, n=20 for OA group). **C, D.**Blocking VCAM-1 with a neutralizing antibody significantly inhibits NsPEFs-ADSCs-EVs-induced up-regulation of RSPO3 at both the protein (n=3) and mRNA (n=6) levels in chondrocytes. Data are presented as mean ± SEM. Statistical significance was determined by unpaired two-tailed Student’s t-test or one-way ANOVA with Tukey’s post-hoc test. *P < 0.05, **P < 0.01, ***P < 0.001, and ****P < 0.0001.

**Tables S1.** **Real-time PCR primer sequences.**

| Gene | Forward primer (5’-3’) | Reverse primer (5’-3’) |
| --- | --- | --- |
| *CD86* | ATGGGGCTCGTATGATTGT | CTTCTTAGGTTTCGGGTG |
| *IL-1β* | GAAATGCCACCTTTTGACAGTG | TGGATGCTCTCATCAGGACAC |
| *IL-10* | GCTCTTACTGACTGGCATGAG | CGCAGCTCTAGGAGCATGTG |
| *CD206* | ATCCACGAGCAAATGTACCTCA | TAGCCAGTTCAGATACCGGAA |
| *Arg1* | CTCCAAGCCAAAGTCCTTAGAG | AGGAGCTGTCATTAGGGACATC |
| *R-spondin3* | CAACCAGCGAGACAAGAACT | TCCAAACCTTTGCTGTCAGAG |
| *Inos* | GTTCTCAGCCCAACAATACAAGA | GTGGACGGGTCGATGTCAC |
| *Gapdh* | TGTTCCTACCCCCAATGTGTCCGTC | CTGGTCCTCAGTGTAGCCCAAGATG |
| *Mmp13* | CTTCTTCTTGTTGAGCTGGACTC | CTGTGGAGGTCACTGTAGACT |
| *Col-2a* | GGGAATGTCCTCTGCGATGAC | CAGGCGCACCATCTCTGAT |
| *Cd163* | ATGGGTGGACACAGAATGGTT | CAGGAGCGTTAGTGACAGCAG |
| *Lgr4* | TACAACTGGCTGGTAACGACC | TTGAGTTCTTTCAACCCAGACAA |
| *Lrp6* | TTGTTGCTTTATGCAAACAGACG | GTTCGTTTAATGGCTTCTTCGC |
| *Lgr5* | CCTACTCGAAGACTTACCCAGT | GCATTGGGGTGAATGATAGCA |
| *Lgr6* | GAGGACGGCATCATGCTGTC | GCTCCGTGAGGTTGTTCATACT |
| *Actb* | CTTCTTCCAGCCTTCCTTCC | GCTGTCACCTTCACCGTCT |
